# Supplementary material for: Gating mechanism of elongating β-ketoacyl-ACP synthases
Source: Nat Commun. 2020 Apr 7;11:1727. doi: 10.1038/s41467-020-15455-x (PMC7138838; doi:10.1038/s41467-020-15455-x)
Supplement: Supplementary file 1 — Supplementary Information [file 41467_2020_15455_MOESM1_ESM.pdf]

Supplementary Information

Gating Mechanism of Elongating β-Ketoacyl-ACP Synthases

Jeffrey T. Mindrebo,[1,3] Ashay Patel,[1] Woojoo E. Kim,[1,] Tony D. Davis,[1] Aochiu Chen,[1] Thomas G. Bartholow,[1] James J. La Clair,[1,3] J. Andrew McCammon[1,2], Joseph P. Noel[1,3,4,*] & Michael D. Burkart[1*]

[1]Department of Chemistry and Biochemistry, University of California, San Diego, 9500 Gilman Drive, La Jolla CA 92093-0358

[2]Department of Pharmacology, University of California, San Diego, 9500 Gilman Drive, La Jolla CA 92093

[3]Jack H. Skirball Center for Chemical Biology and Proteomics, Salk Institute for Biological Studies, La Jolla, CA 92037;

[4] Howard Hughes Medical Institute, Salk Institute for Biological Studies, La Jolla, CA 92037

[*]Corresponding authors: mburkart@ucsd.edu or noel@salk.edu

Table of Contents

Supplementary Figure 1. Chemistry and biology of ketosynthases. 5

Supplementary Figure 2. Illustration of crosslinking reactions between the α-bromo pantetheineamide probe and KS active site cysteine residues. 7

Supplementary Figure 3. Comparison of C16AcpP-FabF and C12AcpP-FabB binding modes. 8

Supplementary Figure 4. Comparison of alkyl chain electron density and substrate•KS interactions in C16AcpP-FabF, C12AcpP-FabB, and C16AcpP-FabB crystal structures. 9

Supplementary Figure 5. Analysis of loop 1 and 2 conservation by multi-sequence alignment (MSA) 10

Supplementary Figure 6. Time-course crosslinking results of of wt FabF and select gating mutants 11

Supplementary Figure 8. Additional G399A and G402A Single time point crosslinking gels of 1a-AcpP (C12αBr) and 1c-AcpP (C2αBr) 13

Supplementary Figure 9. 2a-AcpP (C8Cl) and 2b-AcpP (C3Cl) crosslinking gels. 14

Supplementary Figure 10. 2a-AcpP (C8Cl) time course crosslinking gels. 15

Supplementary Figure 11. 1a-AcpP (C12αBr) time course crosslinking gels. 16

Supplementary Figure 12: Panel of pocket block mutations. 17

Supplementary Figure 13. Acyl-AcpP substrates used in simulations of the acyl-AcpP2•FabB2 and acyl-AcpP2•FabF2 complexes 18

Supplementary Figure 14. a Root mean square fluctuations (RMSFs) of acyl-AcpP2•FabB2 and acyl-AcpP2•FabF2 19

Supplementary Figure 15. Analysis of the substrate binding pocket determined using POVME (POcket Volume Measurer.) 20

Supplementary Figure 16. RMSDs of acyl-AcpP2•FabB2 complexes sampled during cMD simulations. 21

Supplementary Figure 17. RMSDs of acyl-AcpP2•FabF2 complexes sampled during cMD simulations. 22

Supplementary Figure 18. RMSDs of ketosynthase domains of acyl-AcpP2•FabB2 complexes sampled during cMD simulations. 23

Supplementary Figure 19. RMSDs of ketosynthase domains of acyl-AcpP2•FabF2 complexes sampled during cMD simulations. 24

Supplementary Figure 20. RMSDs of AcpPs of acyl-AcpP2•FabB2 complexes sampled during cMD simulations. 25

Supplementary Figure 21. RMSDs of AcpPs of acyl-AcpP2•FabF2 complexes sampled during cMD simulations. 26

Supplementary Figure 22. RMSDs of loop 1 region of FabB within acyl-AcpP2•FabB2 complexes sampled during cMD simulations. 27

Supplementary Figure 23. RMSDs of loop 2 region of FabB within acyl-AcpP2•FabB2 complexes sampled during cMD simulations. 28

Supplementary Figure 24. RMSDs of loop 1 region of FabF within acyl-AcpP2•FabF2 complexes sampled during cMD simulations. 29

Supplementary Figure 25. RMSDs of loop 2 region of FabF within acyl-AcpP2•FabF2 complexes sampled during cMD simulations. 30

Supplementary Figure 26. RMSDs of the nonstandard residue of acyl-AcpP2•FabB2 complexes sampled during cMD simulations..31

Supplementary Figure 27. RMSDs of the nonstandard residue of acyl-AcpP2•FabF2 complexes sampled during cMD simulations..32

Supplementary Figure 28. Root mean square (RMS) fluctuations each residue of loops 1 and 2 of FabB and FabF monomers of the apo-AcpP•KS sampled during MD simulations...............34

Supplementary Figure 29. Root mean square (RMS) fluctuations each residue of loop 1 of FabB and FabF monomers of the acyl-AcpP•KS sampled during MD simulations. 35

Supplementary Figure 30. Root mean square (RMS) fluctuations each residue of loop 2 of FabB and FabF monomers of the acyl-AcpP2•KS2 sampled during MD simulations...............36

Supplementary Figure 31. Ramachandran plots of the GFGG motifs of FabB or FabF simulated in various states. 37

Supplementary Figure 32. Ramachandran plots of the key residues of loop 2 of FabB or FabF simulated in various states...38

Supplementary Figure 33. Primers for generating FabF mutants..39

Supplementary Figure 34. Table 1 Refinement statistics from structures solved in this work..40

Supplementary Figure 35. Electron density maps of reported structures...............................41

Supplementary Figure 36. Native contact analysis of MD simulations data of acyl-AcpP2•FabB2 (10:0-AcpP2•FabB2, 12:0-AcpP2•FabB2, 16:0-AcpP2•FabB2). 42

Supplementary Figure 37. Native contact analysis of MD simulations data of acyl-AcpP2•FabF2 (10:0-AcpP2•FabF2, 12:0-AcpP2•FabF2, 16:0-AcpP2•FabF2)...........................43

Supplementary Figure 38. Analysis of contacts between active site residues and substrate of acyl-AcpP•FabF complexes sampled computationally...44

Supplementary Figure 39. Analysis of contacts between active site residues and substrate of acyl-AcpP•FabB complexes sampled computationally. 45

Supplementary Note 1. C16AcpP-FabB complex 46

Supplementary Note 2. C12AcpP-FabF complex 47

Supplementary Note 3. C16AcpP-FabF delineates a potential minor conformation for the C16 alkyl chain 48
Supplementary Note 4. Comparison of FabF and FabB structural features 50
Supplementary Note 5. Time resolved analysis of AcpP-KS interfaces 48
Supplementary Note 6. Substrate interaction analysis in AcpP•FabF simulations 53
Supplementary Note 7. Substrate interaction analysis in AcpP•FabB simulations 54
Supplementary Note 8. General synthetic methods. 55
Supplementary Figure 40. NMR characterization of S2a 59
Supplementary Figure 41. NMR characterization of S2b 60
Supplementary Figure 42. NMR characterization of S3a 61
Supplementary Figure 43. NMR characterization of S3b 62
Supplementary References........ 63

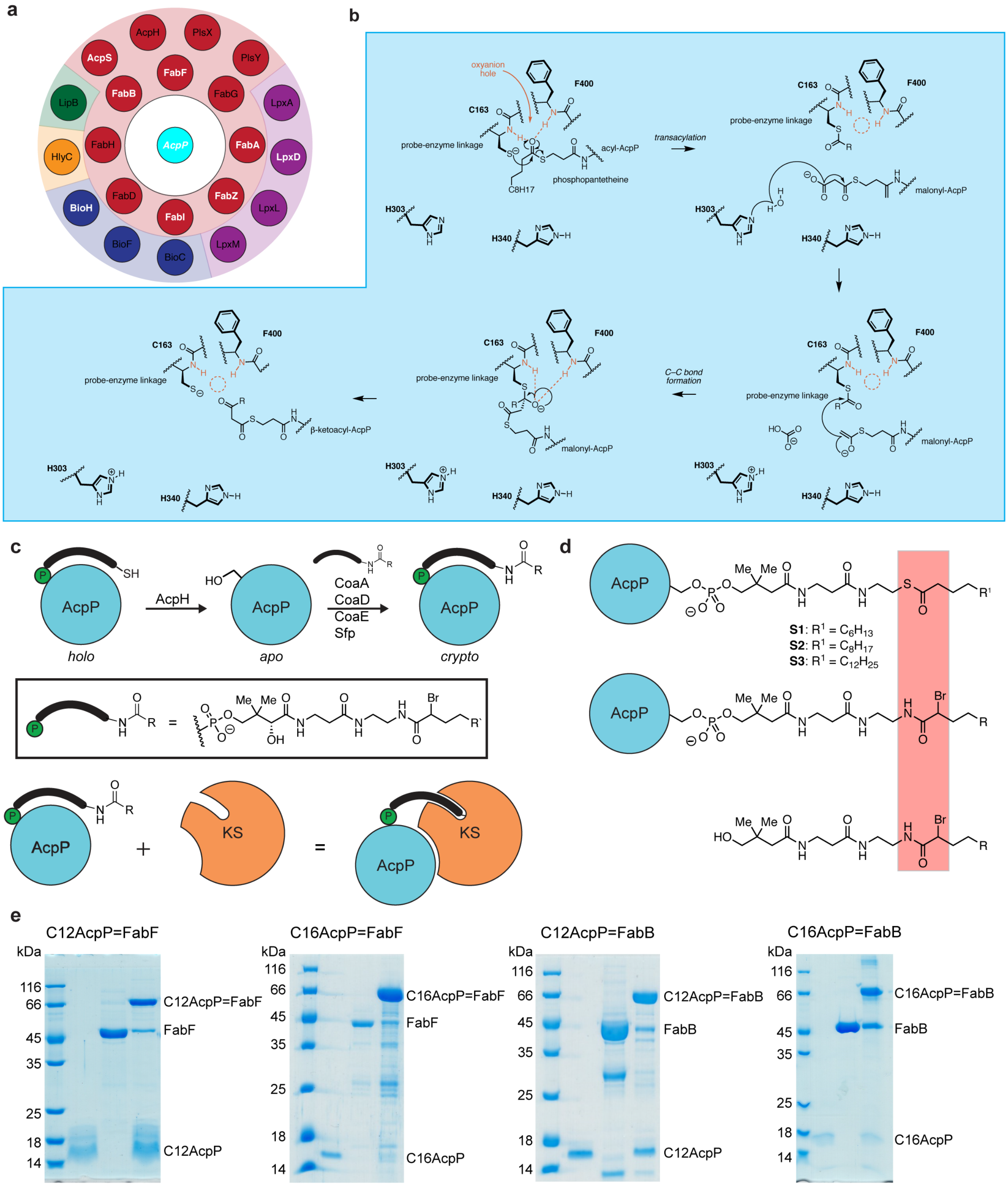

Supplementary Figure 1. Chemistry and biology of ketosynthases. a. ACP "Interactome" in *E. coli*. Proteins are colored by biosynthetic pathway (red: fatty acid biosynthesis, purple: lipid A biosynthesis, Blue: biotin biosynthesis, yellow: hemolysin activation, green: lipoyl activation of lipoate-dependent enzymes. **b.** Proposed reaction mechanism of elongating β-ketoacyl-AcpP-synthase FabB (KASI) and FabF (KASII). Top row of mechanism shows the transacylation half-reaction, while the bottom portion illustrates the condensation half-reaction. **c.** Crosslinking workflow scheme demonstrating apofication and loading of ACP with crosslinking probes to

produce *crypto*-ACP, which is then mixed with partner protein to produce a crosslinked complex. **d.** Comparison of acyl-AcpP to crypto-AcpP loaded with substrate or crosslinking substrate mimetic, respectively. **S1**, **S2**, and **S3** indicate the acyl-AcpPs subjected to simulations as constituents of acyl-AcpP•KS complexes reported herein **e.** Crosslinking gels of C12AcpP-FabF, C16AcpP-FabF, C12AcpP-FabB, and C16AcpP-FabB that were used to verify the formation of crosslinked complex before purification and crystallization.

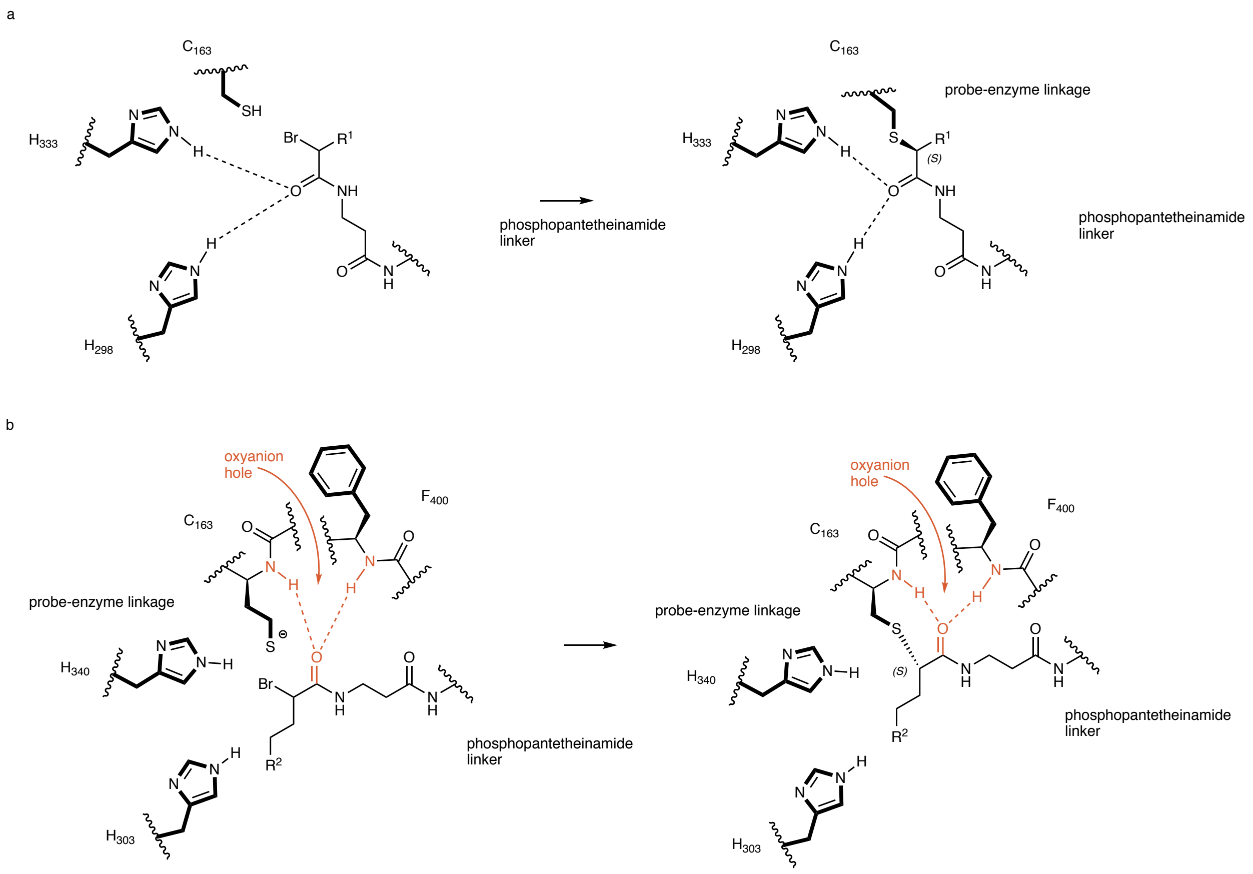

Supplementary Figure 2. Illustration of crosslinking reactions between the α-bromo pantetheineamide probe and KS active site cysteine residues. Hypothetical crosslinking reactions of C16αBr-crypto-AcpP and C12αBr-crypto-AcpP with FabF and FabB. C16AcpP-FabF (**a**) and C12AcpP-FabB (**b**). $R^1 = C_{12}H_{25}$ and $R^2 = C_8H_{17}$

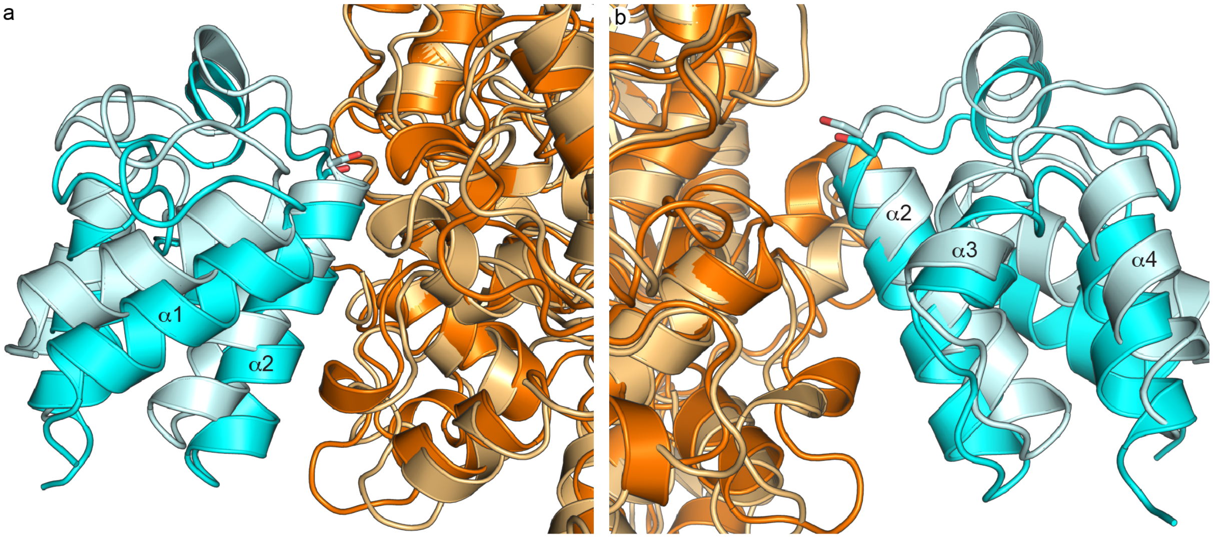

Supplementary Figure 3. Comparison of C16AcpP-FabF and C12AcpP-FabB binding modes. **a. b.** Superposition of C12AcpP=FabB (AcpP shown as light cyan) with C16AcpP=FabB (AcpP shown as cyan) shown from two 180° related views demonstrating differences in the angle and conformation of AcpP engagement with each KS interface.

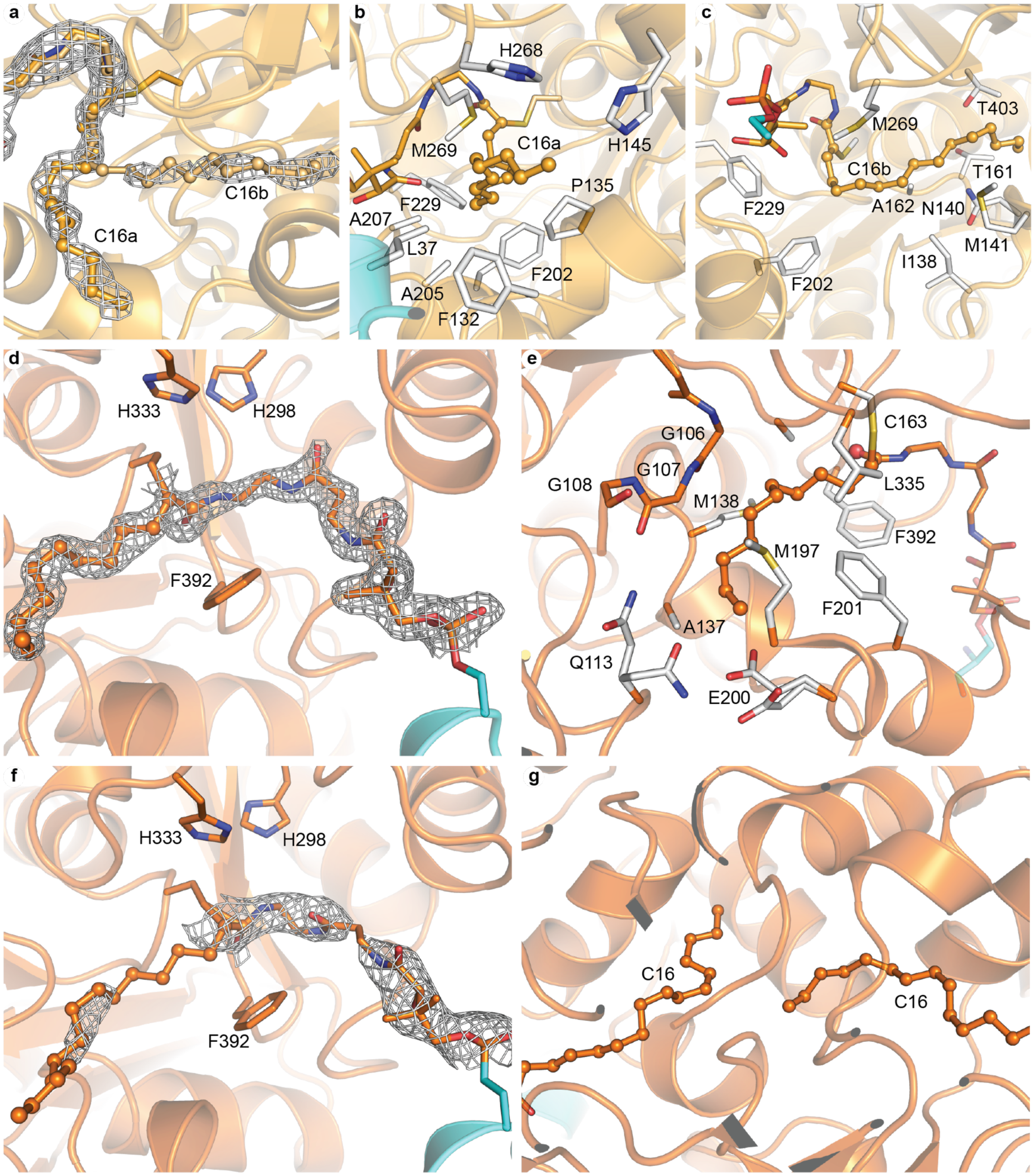

Supplementary Figure 4. Comparison of alkyl chain electron density and substrate·KS interactions in C16AcpP-FabF, C12AcpP-FabB, and C16AcpP-FabB crystal structures. AcpP is shown as cyan in all panels. FabF is shown in light orange and FabB is shown in bright orange. **a.** Electron density of C16 alkyl chain in conformations a (C16a) and b (C16b) from the C16AcpP-FabF structure contoured with 2Fo-Fc maps at 1.0 σ **b.** All FabF residues within 4 Å of alkyl chain conformation a (C16a). **c.** All residues within 4 Å of alkyl chain conformation b (C16b). **d.** Electron density of C12 alkyl chain from C12AcpP-FabB structure contoured at 1.0 σ. **e.** All FabB residues within 4 Å of the C12 alkyl chain substrate analog. **f.** Electron density of C16PPant probe from the C16AcpP-FabB structure contoured at 1.0 σ. **g.** Attempts to model a C16 probe reveal a constricted active site that places alkyl chains bound within the active site of both KS monomers in close proximity to one another and in unfavorable conformations

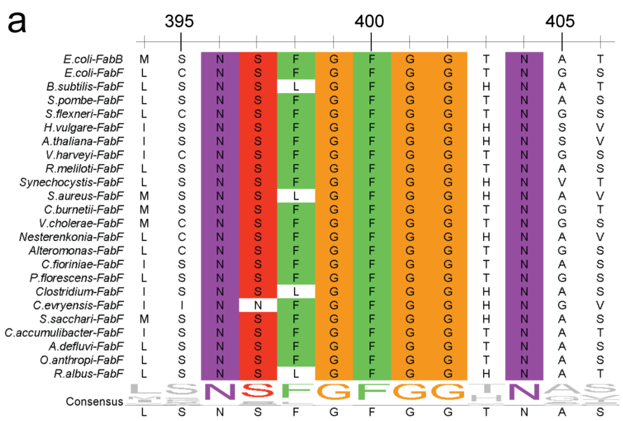

Supplementary Figure 5. Analysis of loop 1 and 2 conservation by multi-sequence alignment (MSA) **a.** MSA alignment of FabB and 100 FabF orthologues comparing the Loop 1 GFGG β-turn region and surrounding conserved residues (FabF numbering). **b**. MSA Alignment of FabB and 100 FabF orthologues comparing the loop 2 sequence and surrounding conserved residues (FabF numbering).

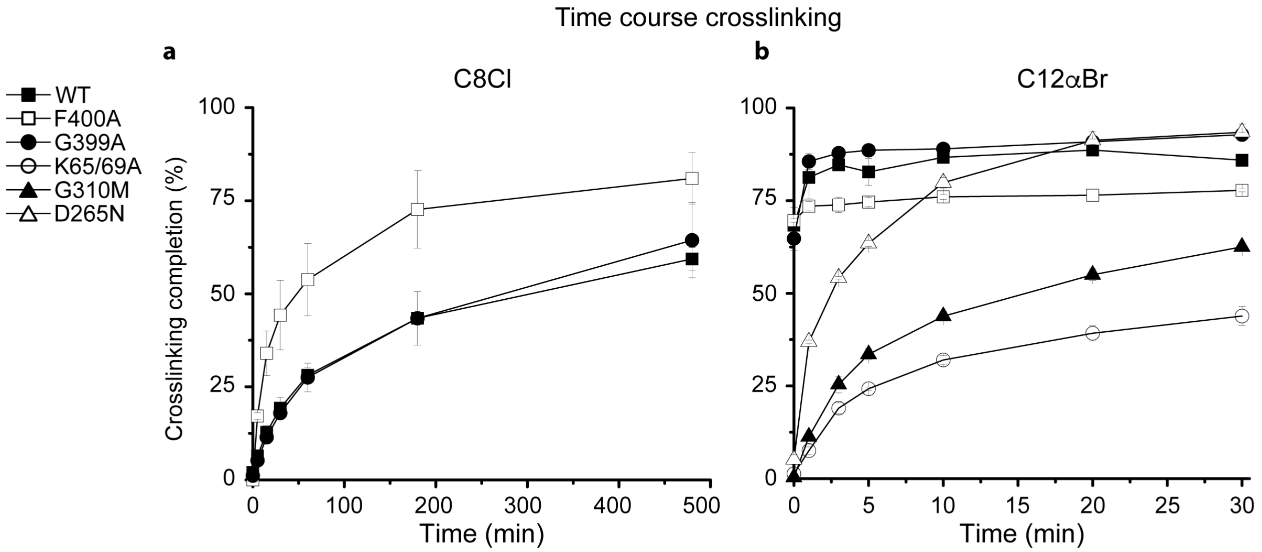

Supplementary Figure 6. Time-course crosslinking results of of wt FabF and select gating mutants. C8-chloroacrylate-AcpP (**a**) and C12-α-Bromo-AcpP (**b**). Results in panel **a** indicate that the F400A gate removal mutant crosslinks faster than the wt enzyme using C8Cl probes and G399 crosslinks similarly to wt FabF. Results using the C12αBr probes in panel **b** show that the gating mutant K65A/K69A, pocket-block mutant G310M, and destabilization mutant D265N all crosslinked at slower rates as compared to wt FabF, G399A, and F400A. G310M and K65A/K69A show the most pronounced reduction in crosslinking rate. All gel-based time-course crosslinking assays were run as biologically independent experiments (n = 3) and analyzed by densitometry using ImageJ. The data are plotted as the average crosslinking efficiency at each respective time point and error bars are represented as standard deviation (± SD). The scanned images of associated gels can be found in Supplementary Figs. 10 and 11. wt FabF (black square), F400A (white square), G399A (black circle), K65A/K69A (white circle), G310M (black triangle), D265N (white triangle). Source data for all experiments are provided as a Source Data file.

a

1c-AcpP (C2αBr)

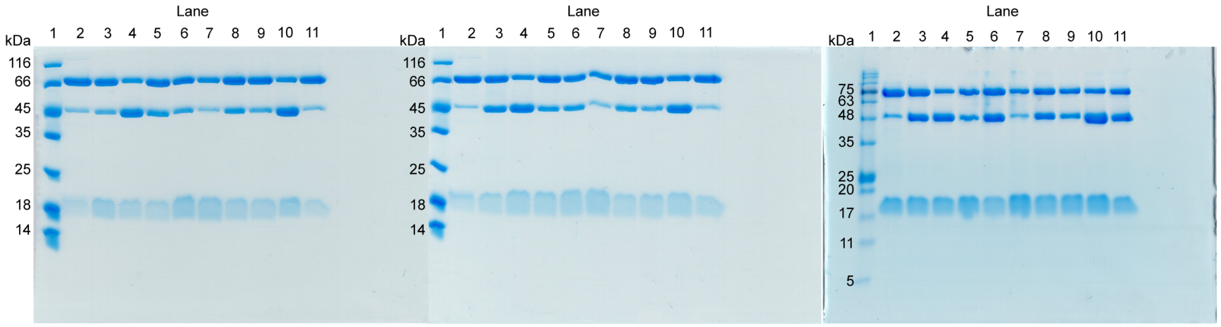

b

1a-AcpP (C12αBr)

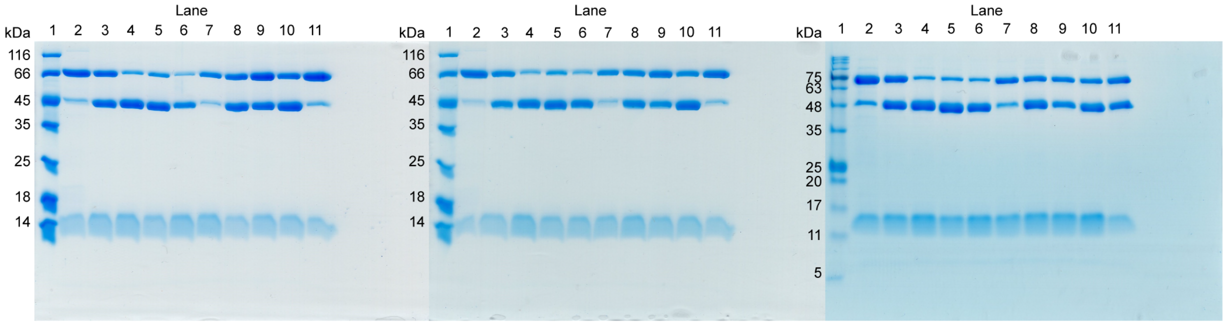

Supplementary Figure 7. Single time point crosslinking gels of 1a-AcpP (C12αBr) and 1c-AcpP (C2αBr). The gels shown above are the full scans of the single time point crosslinking gels of **1c**-AcpP (C2αBr) and **1a**-AcpP (C12αBr) with FabF gating mutants from three independent biological experiments (n = 3). More specifically, these gels represent the raw crosslinking gel data used for the data reported in panels **d** and **e** of Figure 6 in the main text. **a.** Biological independent experiments (n = 3) of **1c**-AcpP (C2αBr) crosslinking experiments with FabF mutant. Lane 1 - ladder, lane 2 – wt FabF, lane 3 – K65E, lane 4 – K65A/K65E, lane 5 – G310F, lane 6 – G310M, lane 7 – F400A, lane 8 – D265A, lane 9 – D265N, lane 10 – G402A, lane 11 -G399A. **b.** Biological independent experiments (n = 3) of **1a**-AcpP (C12αBr) crosslinking with FabF mutant panel. Lane 1 - ladder, lane2 – wt FabF, lane 3 – K65E, lane 4 – K65A/K65E, lane 5 – G310F, lane 6 – G310M, lane 7 – F400A, lane 8 – D265A, lane 9 – D265N, lane 10 – G402A, lane 11 -G399A.

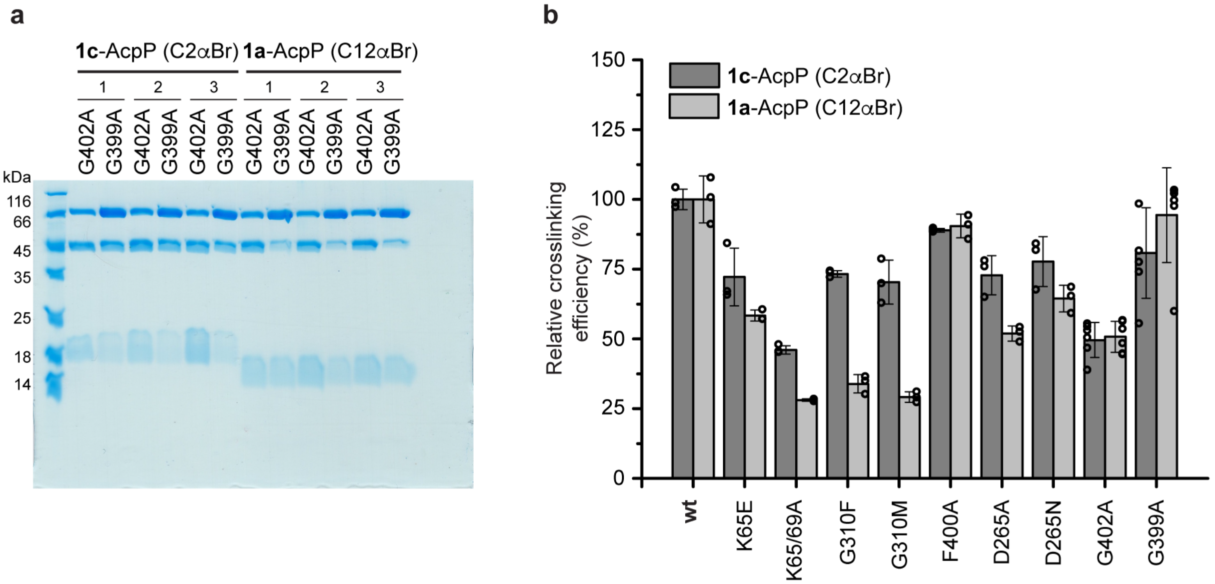

Supplementary Figure 8. Additional G399A and G402A single time point crosslinking gels of 1a-AcpP (C12αBr) and 1c-AcpP (C2αBr). **a.** The gel shown above is the full scan of single time point crosslinking analysis of **1c**-AcpP (C2αBr) and **1a**-AcpP (C12αBr) with FabF gating mutants G399A and G402A performed separately from the original experiments due to an outlier measurement in G399A (see lowest open circle in G399A in graph found in panel **b**). These data represent three additional independent biological experiments (n = 3) for G399A and G402A with **1c**-AcpP (C2αBr) and **1a**-AcpP (C12αBr). **b.** Densitometric analysis of single time-point crosslinking efficiency of **1c**-AcpP (C2αBr-AcpP) and **1a**-AcpP (C12αBr-AcpP) with FabF mutants including the three additional biologically independent data points derived from the G399A and G402A crosslinking assay shown in panel **a**. All crosslinking experiments from were performed as biologically independent experiments (n = 3), except for G399A and G402A which were done in sextuplicate (n = 6). All data represented in **b** are the average crosslinking efficiency of each mutant normalized to the average of wt FabF crosslinking efficiency. The error bars in d and e are represented as standard deviation (± SD) and the individual normalized measurements from each independent experiment are overlaid on top of the associated bar plot as open circles. Source data for all experiments are provided as a Source Data file.

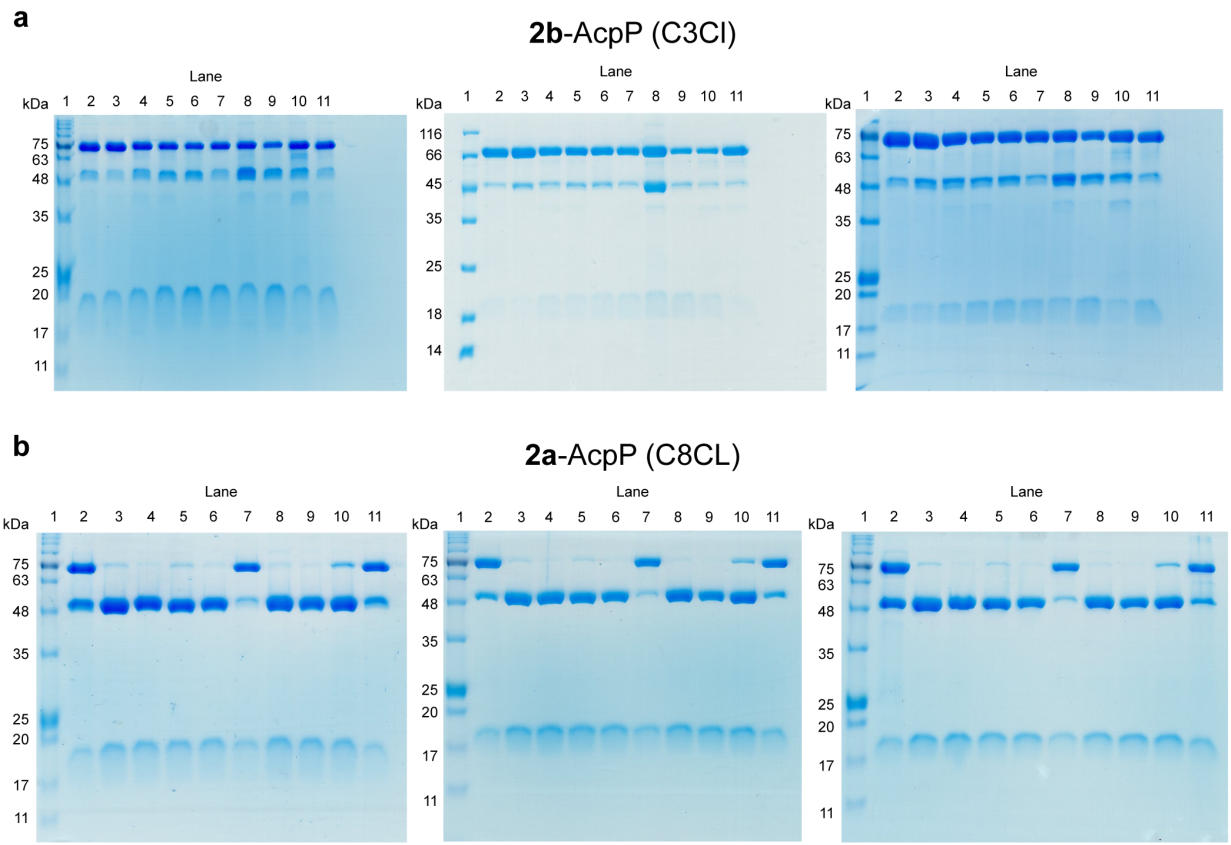

Supplementary Figure 9. 2a-AcpP (C8Cl) and 2b-AcpP (C3Cl) crosslinking gels. The gels shown above are the full scans of the single time point crosslinking gels of **2b**-AcpP (C3Cl) and **2a**-AcpP (C8Cl) with FabF gating mutants from three independent biological experiments (n = 3). More specifically, these gels represent the raw crosslinking gel data used for the data reported in panels **c** and **f** of Figure 6 in the main text. **a.** Biological independent experiments (n = 3) of **2b**-AcpP (C3Cl) crosslinking experiments with FabF mutant panel. Lane 1 - ladder, lane2 – wt FabF, lane 3 – K65E, lane 4 – K65A/K65E, lane 5 – G310F, lane 6 – G310M, lane 7 – F400A, lane 8 – D265A, lane 9 – D265N, lane 10 – G402A, lane 11 -G399A. **b.** Biological independent experiments (n = 3) of **2a**-AcpP (C8Cl) crosslinking experiments with FabF mutant panel. Lane 1 - ladder, lane2 – wt FabF, lane 3 – K65E, lane 4 – K65A/K65E, lane 5 – G310F, lane 6 – G310M, lane 7 – F400A, lane 8 – D265A, lane 9 – D265N, lane 10 – G402A, lane 11 - G399A. All reactions were run in parallel for 8 hours then quenched with SDS loading buffer and boiled.

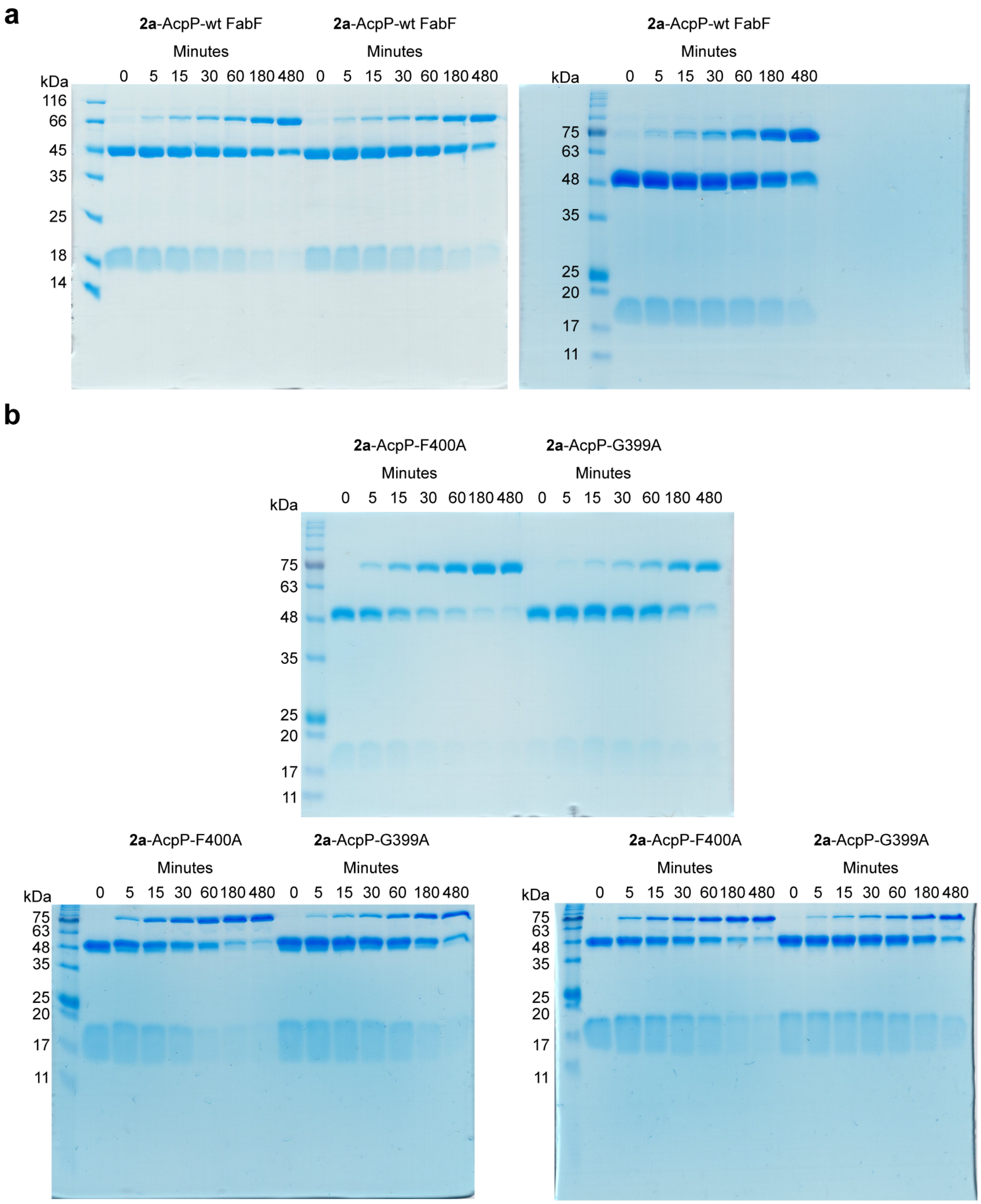

Supplementary Figure 10. 2a-AcpP (C8Cl) time course crosslinking gels. The gels shown above are the full scans of the time course crosslinking gels of **2a**-AcpP (C8Cl) with FabF gating mutants run as three independent biological experiments (n = 3). More specifically, these gels represent the raw crosslinking gel data used for the data reported in supplementary figure 5a. **a.** Biological independent experiments (n = 3) of **2a**-AcpP crosslinking with wt FabF. **b.** Biological independent experiments (n = 3) of **2a**-AcpP crosslinking with FabF mutants F400A and G399A. All reactions were run in parallel and quenched at their respective timepoints by boiling after treatment with SDS loading buffer.

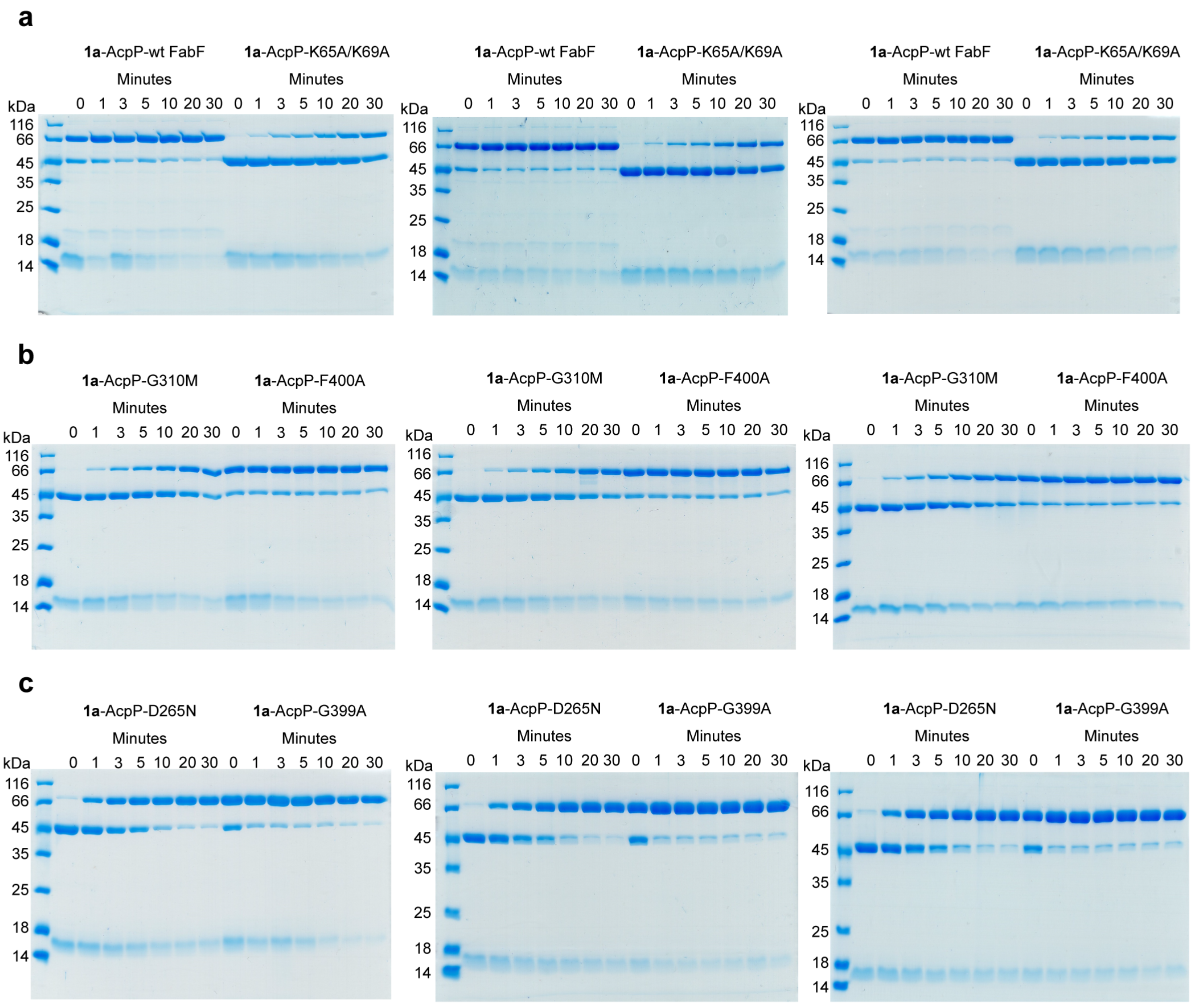

Supplementary Figure 11. 1a-AcpP (C12αBr) time course crosslinking gels. The gels shown above are the full scans of the time course crosslinking experiment gels of **1a**-AcpP (C12αBr) with FabF gating mutants run as three independent biological experiments (n = 3). More specifically, these gels represent the raw crosslinking gel data used for the data reported in supplementary figure 5b. **a.** Biological independent experiments (n = 3) of **1a**-AcpP crosslinking with FabF wt and K65A/K69A FabF interface mutant. **b.** Biological independent experiments (n = 3) of **1a**-AcpP crosslinking with FabF mutants G310M and F400A. **c.** Biological independent experiments (n = 3) of **1a**-AcpP crosslinking with FabF mutants D265N and G399A. All reactions were run in parallel and quenched at their respective timepoints by boiling after treatment with SDS loading buffer.

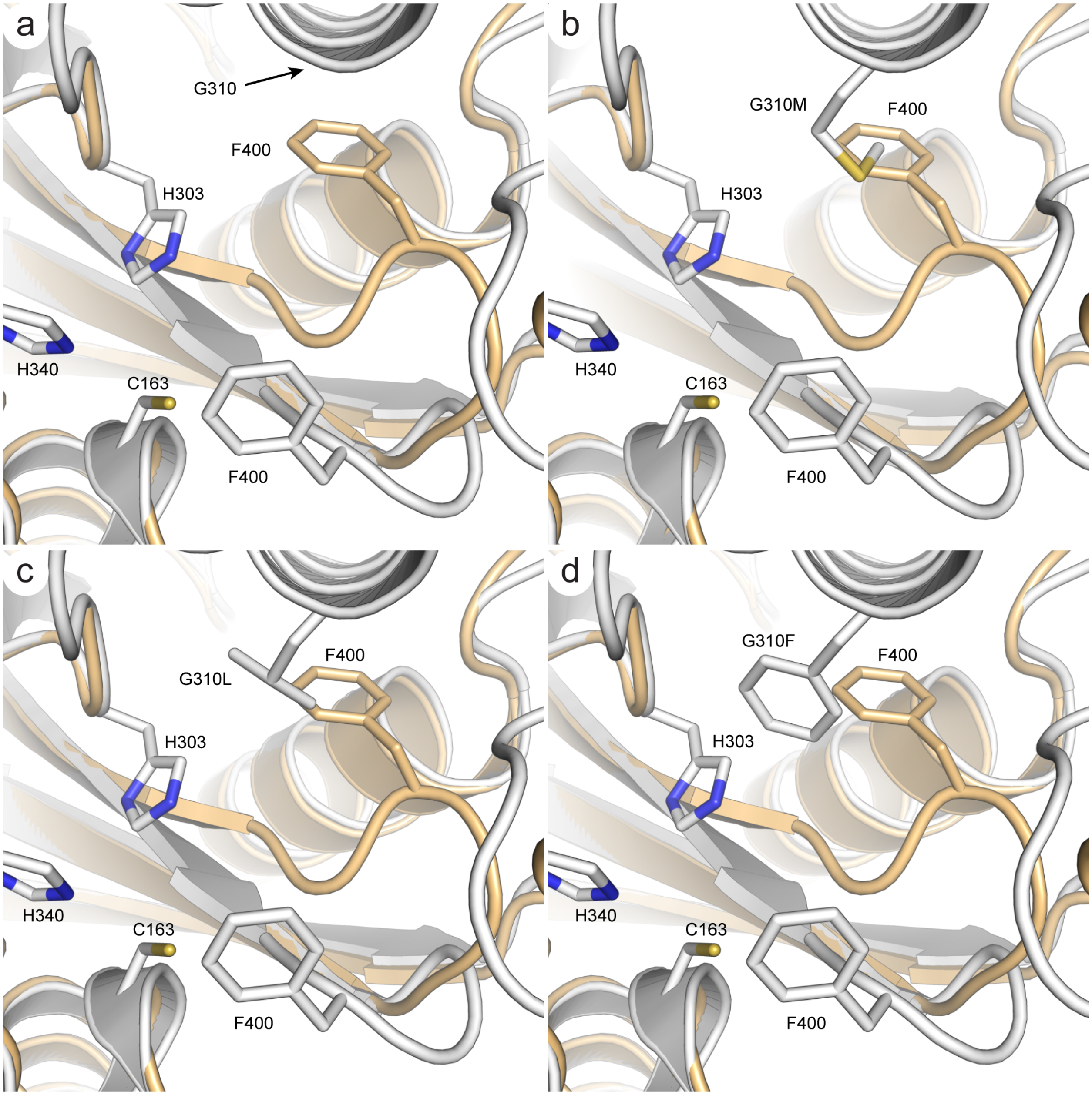

Supplementary Figure 12: Panel of pocket block mutations. Figures were generated by mutating Gly310 into bulky hydrophobic residues in PyMOL. G310, when replaced by larger amino acids, occupies the same pocket as Phe400 in the open conformation (light orange) and would likely inhibit transitions of loop 1 from the closed conformation (light-grey) (PDB: 2GFW) to the open conformation (light orange) (C16AcpP-FabF, PDB: 6OKG). **a.** wt G310 FabF. **b.** G310M. **c.** G310F. **d.** G310L.

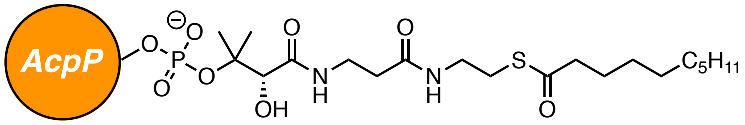

decanoyl-AcpP

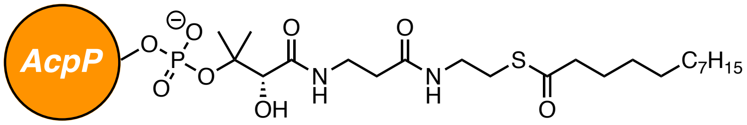

dodecanoyl-AcpP

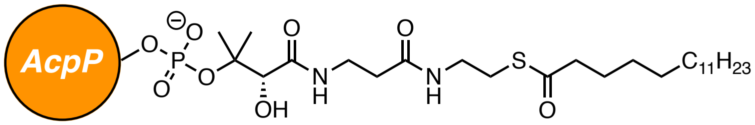

hexadecanoyl-AcpP

Supplementary Figure 13. Acyl-AcpP substrates used in simulations of the acyl-AcpP2•FabB2 and acyl-AcpP2•FabF2 complexes.

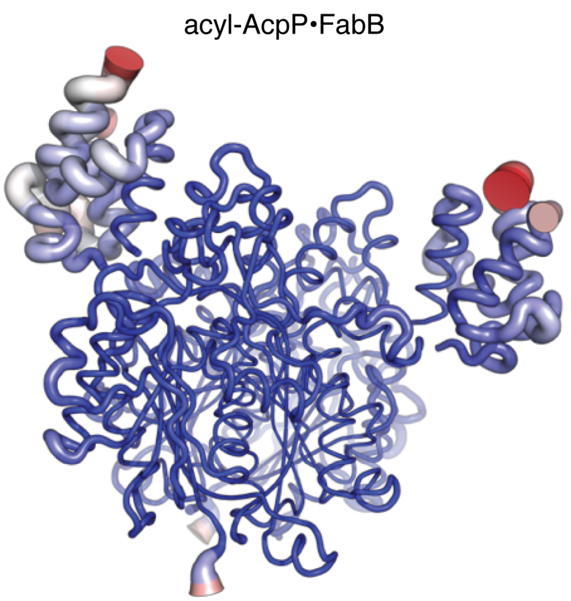

Supplementary Figure 14. Root mean square fluctuations (RMSFs) of acyl-AcpP2·FabB2 and acyl-AcpP2·FabF2 (determined from simulations) “mapped” onto the x-ray crystal structure of the crosslinked acyl-AcpP-FabB and acyl-AcpP-FabF complexes. Larger (per residue) backbone RMSF values correspond to a thicker “sausage”; color range (blue to white to red) illustrates (per residue) side chain RMSFs with the blue-to-red color range indicating small-to-large RMSF values.

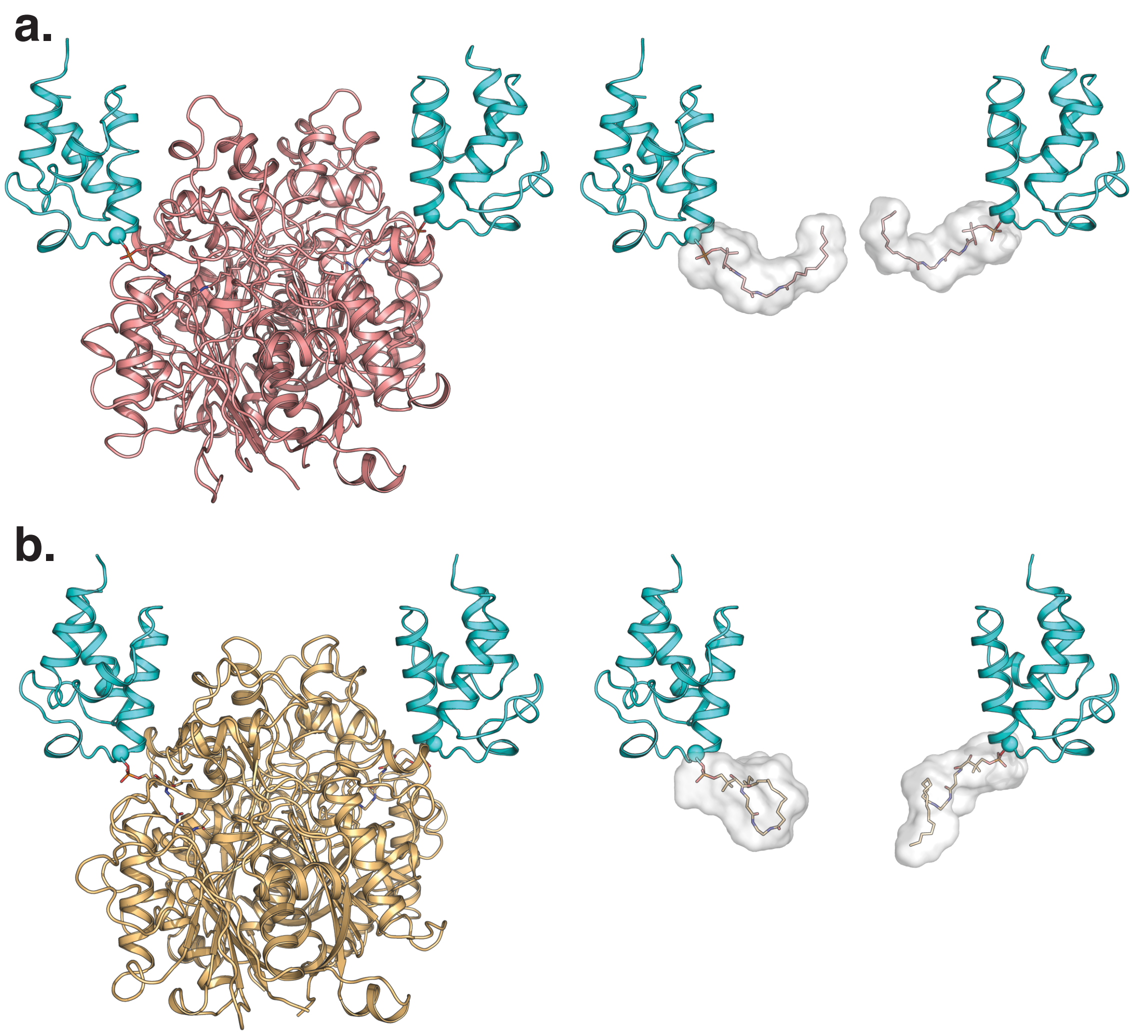

Supplementary Figure 15. Analysis of the substrate binding pocket determined using POVME (POcket Volume Measurer.) a, acyl-AcpP•FabB. b,acyl-AcpP-FabF.

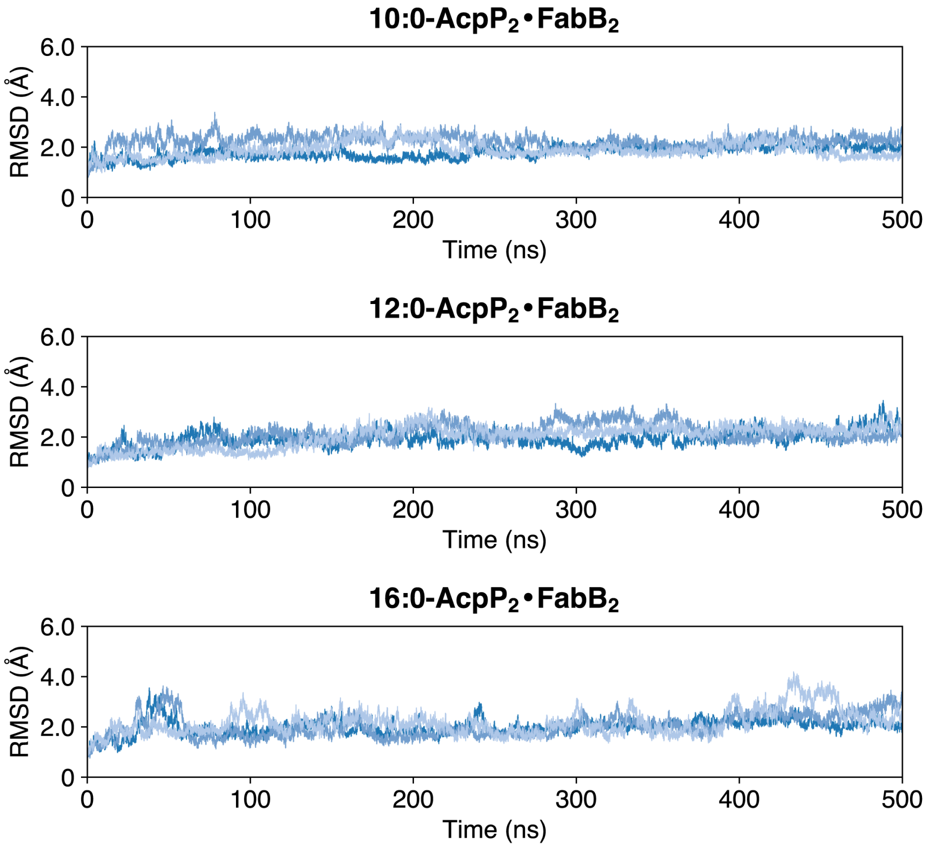

Supplementary Figure 16. RMSDs of acyl-AcpP2•FabB2 complexes sampled during cMD simulations. Each blue curve represents an independent 500 ns simulation. Plots were generated by taking every 10th snapshot of the simulation data.

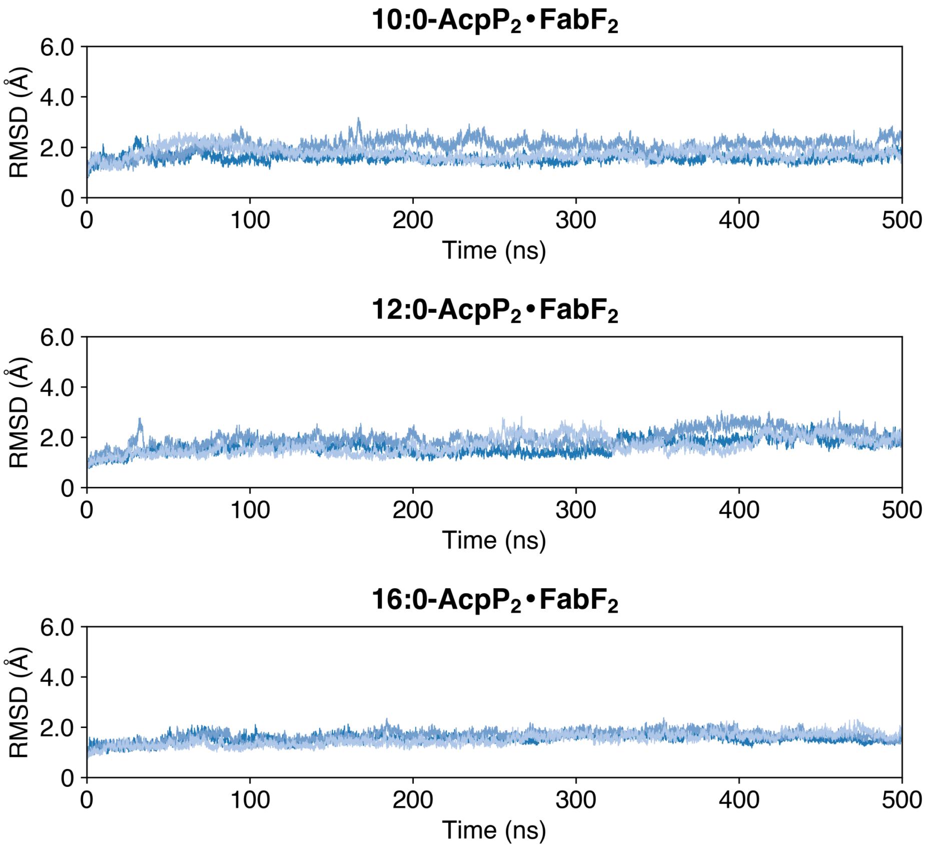

Supplementary Figure 17. RMSDs of acyl-AcpP2•FabF2 complexes sampled during cMD simulations. Each blue curve represents an independent 500 ns simulation. Plots were generated by taking every 10th snapshot of the simulation data.

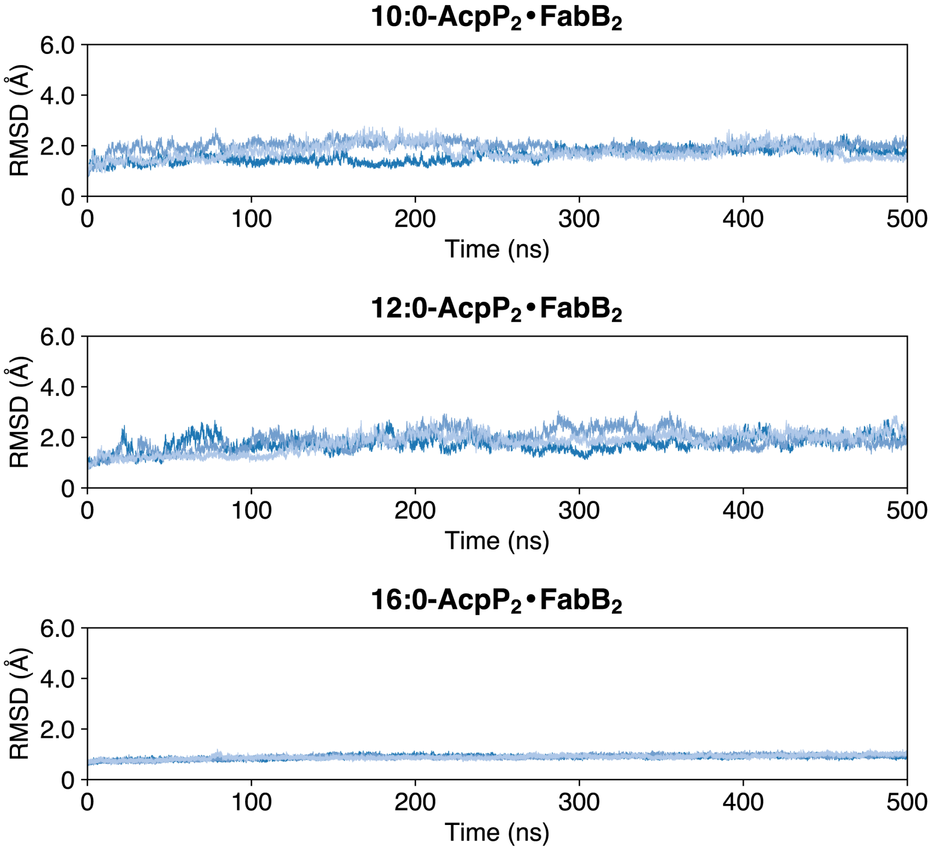

Supplementary Figure 18. RMSDs of ketosynthase domains of acyl-AcpP2•FabB2 complexes sampled during cMD simulations. Each red curve represents an independent 500 ns simulation. Plots were generated by taking every 10th snapshot of the simulation data.

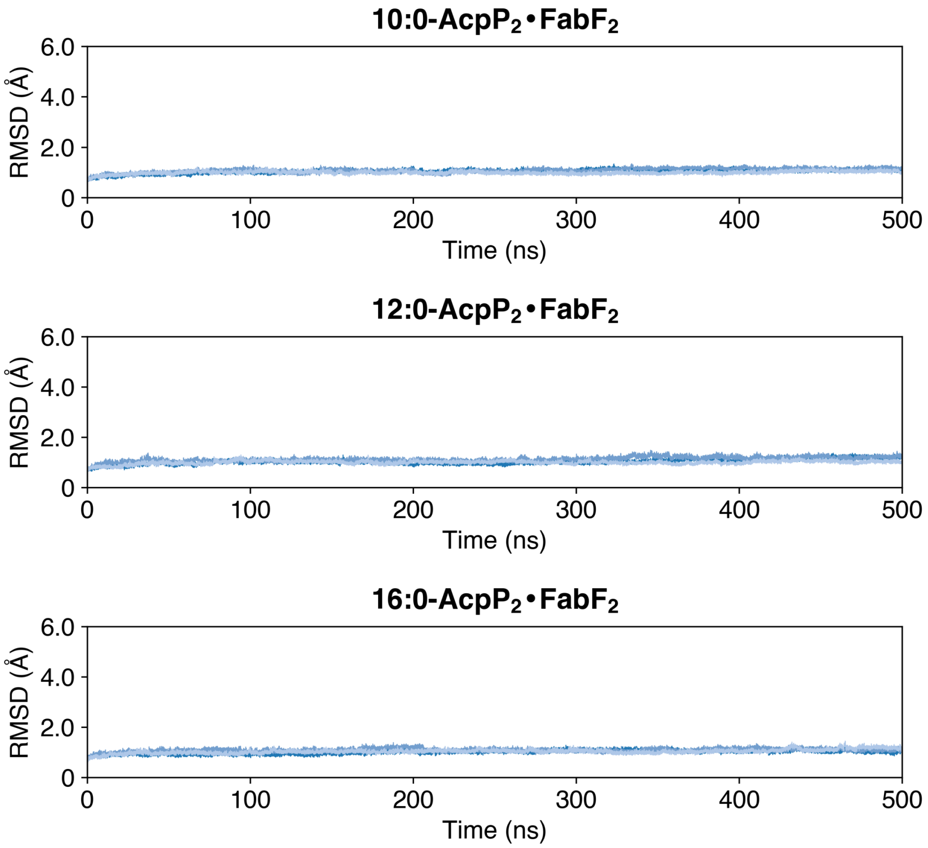

Supplementary Figure 19. RMSDs of ketosynthase domains of acyl-AcpP2•FabF2 complexes sampled during cMD simulations. Each red curve represents an independent 500 ns simulation. Plots were generated by taking every 10th snapshot of the simulation data.

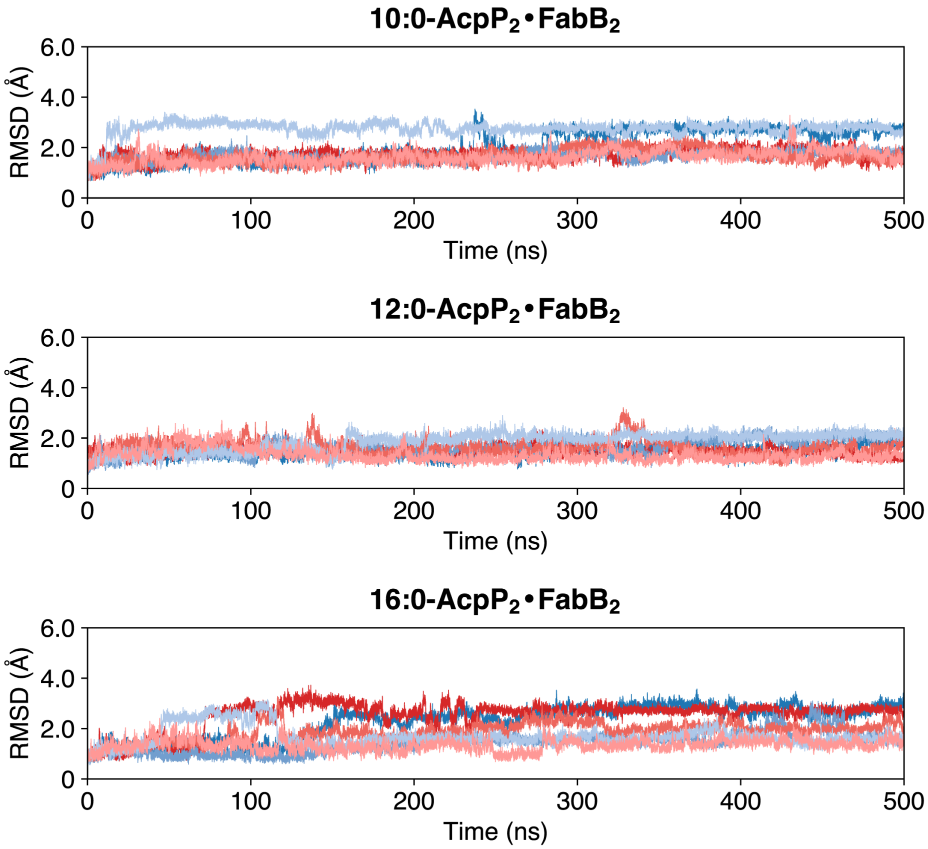

Supplementary Figure 20. RMSDs of AcpPs of acyl-AcpP2·FabB2 complexes sampled during cMD simulations. Each blue (AcpP1) and red (AcpP2) curve represents data from an independent 290 ns simulation. Plots were generated by taking every 10th snapshot of the simulation data.

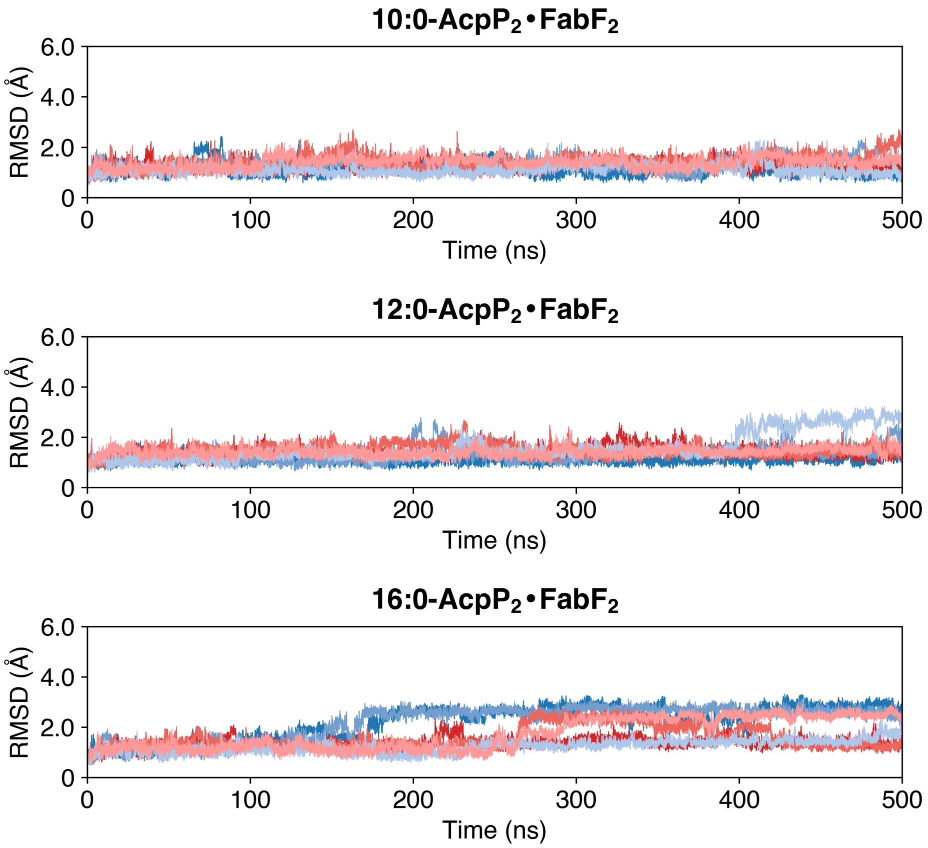

Supplementary Figure 21. RMSDs of AcpPs of acyl-AcpP2·FabF2 complexes sampled during cMD simulations. Each blue (AcpP1) and red (AcpP2) curve represents data from an independent 500 ns simulation. Plots were generated by taking every 10th snapshot of the simulation data.

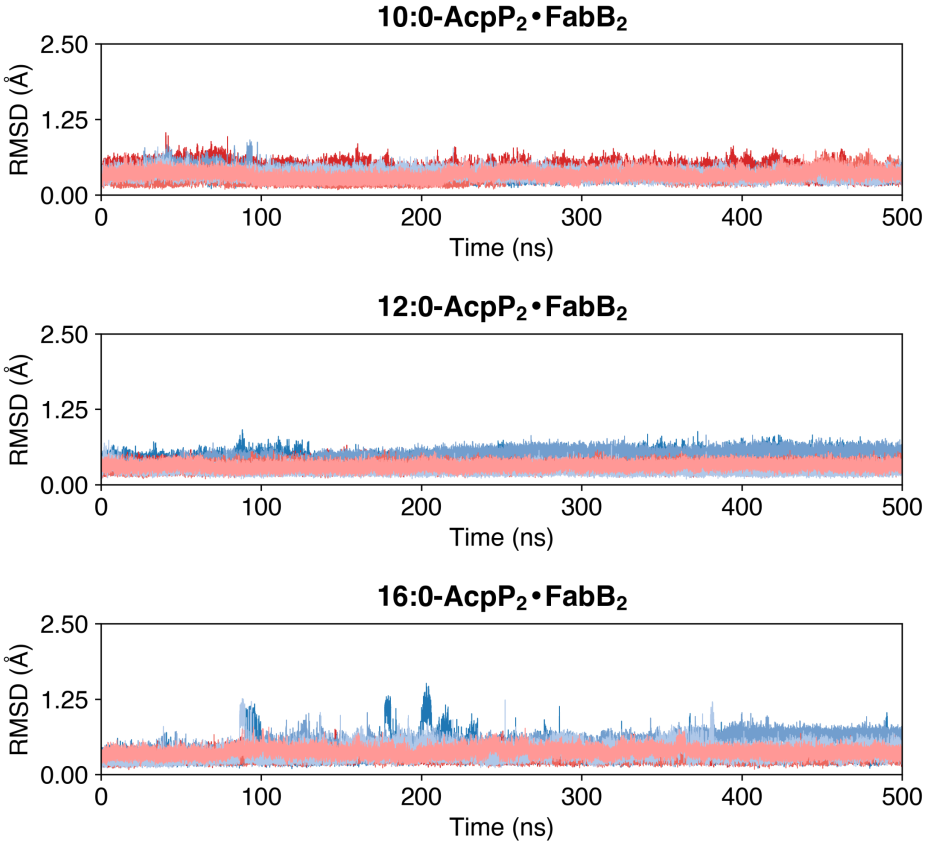

Supplementary Figure 22. RMSDs of loop 1 region of FabB within acyl-AcpP2•FabB2 complexes sampled during cMD simulations. Each blue (loop 1 of FabB1) and red (loop 1 of FabB2) curve represents data from an independent 500 ns simulation.

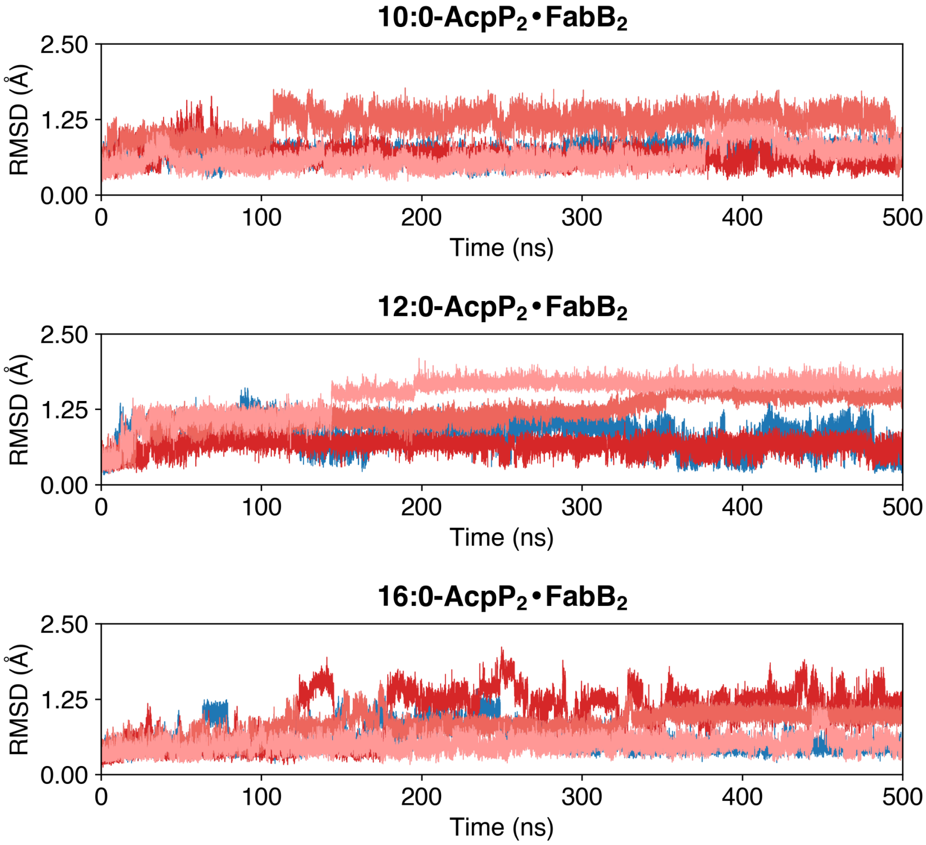

Supplementary Figure 23. RMSDs of loop 2 region of FabB within acyl-AcpP2·FabB2 complexes sampled during cMD simulations. Each blue (loop 1 of FabB1) and red (loop 1 of FabB2) curve represents data from an independent 500 ns simulation.

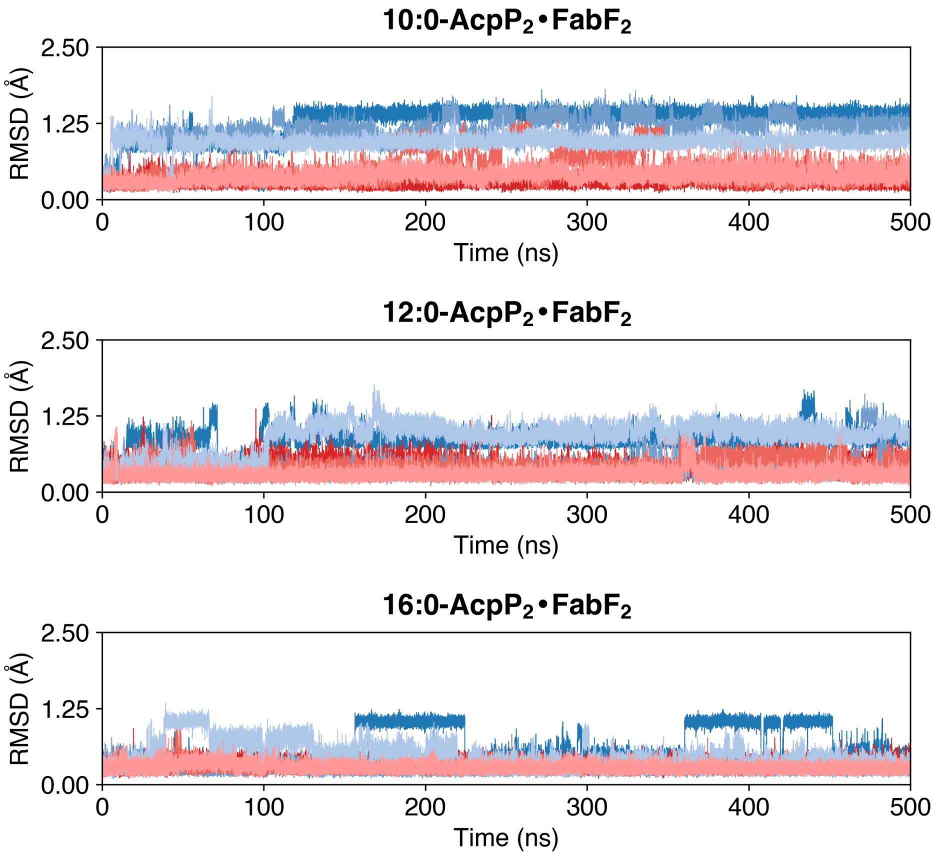

Supplementary Figure 24. RMSDs of loop 1 region of FabF within acyl-AcpP2·FabF2 complexes sampled during cMD simulations. Each blue (loop 1 of FabF1) and red (loop 1 of FabF2) curve represents data from an independent 500 ns simulation.

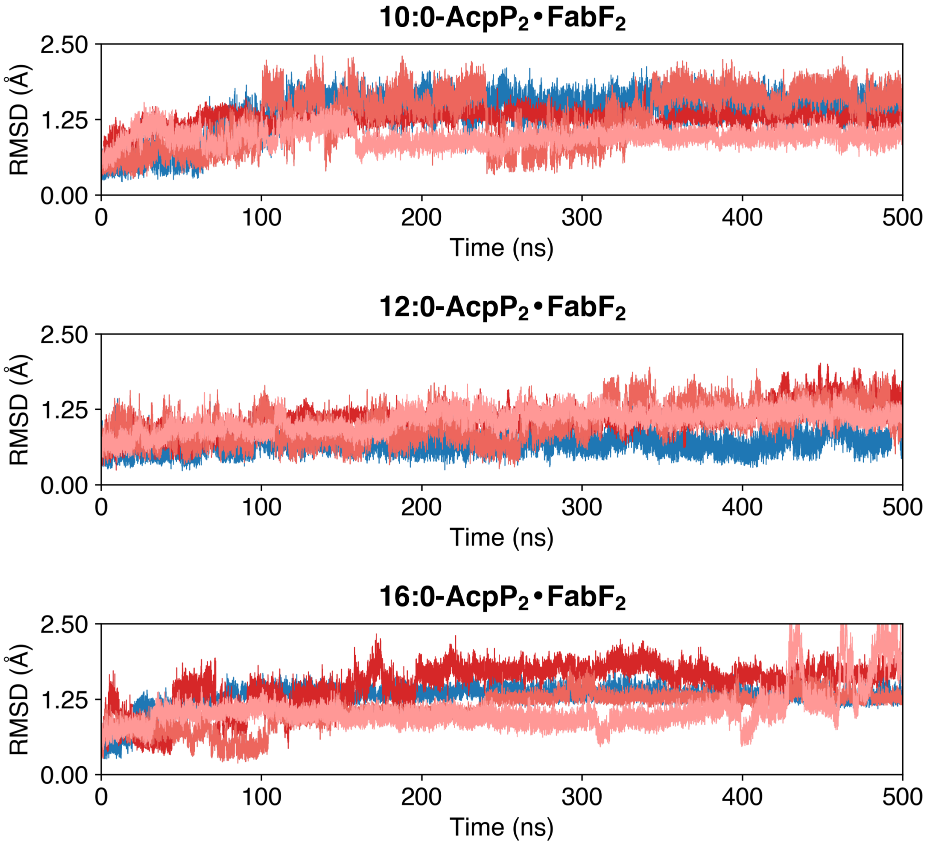

Supplementary Figure 25. RMSDs of loop 2 region of FabF within acyl-AcpP2·FabF2 complexes sampled during cMD simulations. Each blue (loop 2 of FabB1) and red (loop 2 of FabB2) curve represents data from an independent 500 ns simulation.

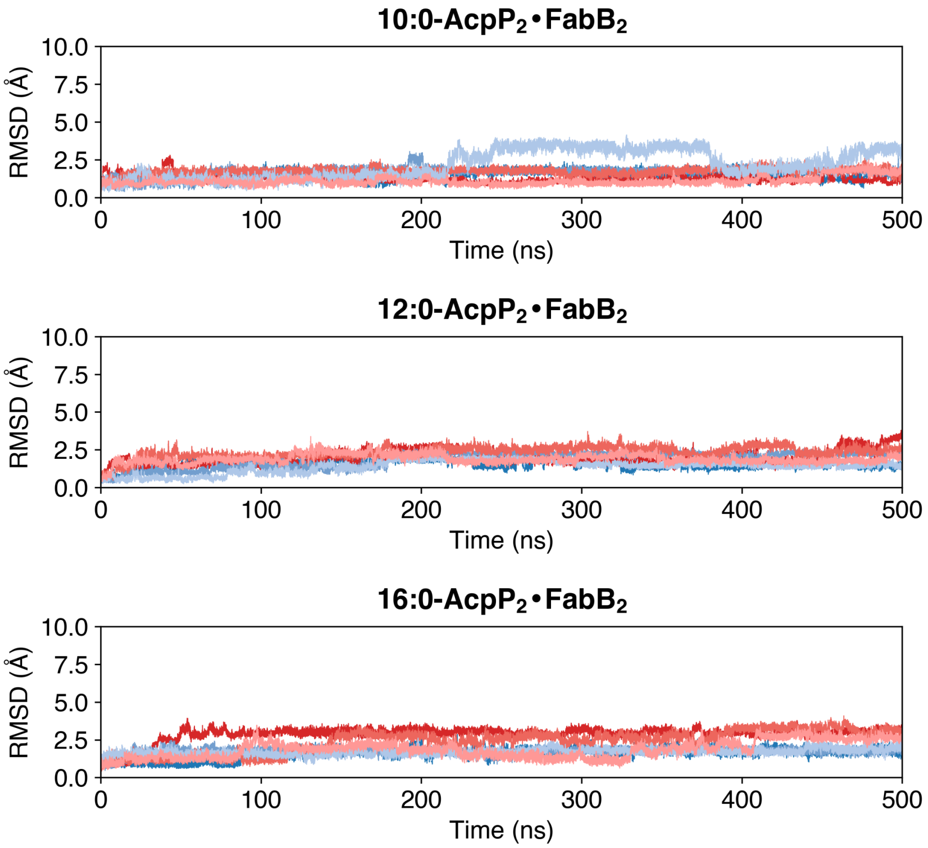

Supplementary Figure 26. RMSDs of the nonstandard residue of acyl-AcpP2·FabB2 complexes sampled during cMD simulations. Each blue (FabB1) and red (FabB2) curve represents data from an independent 500 ns simulation.

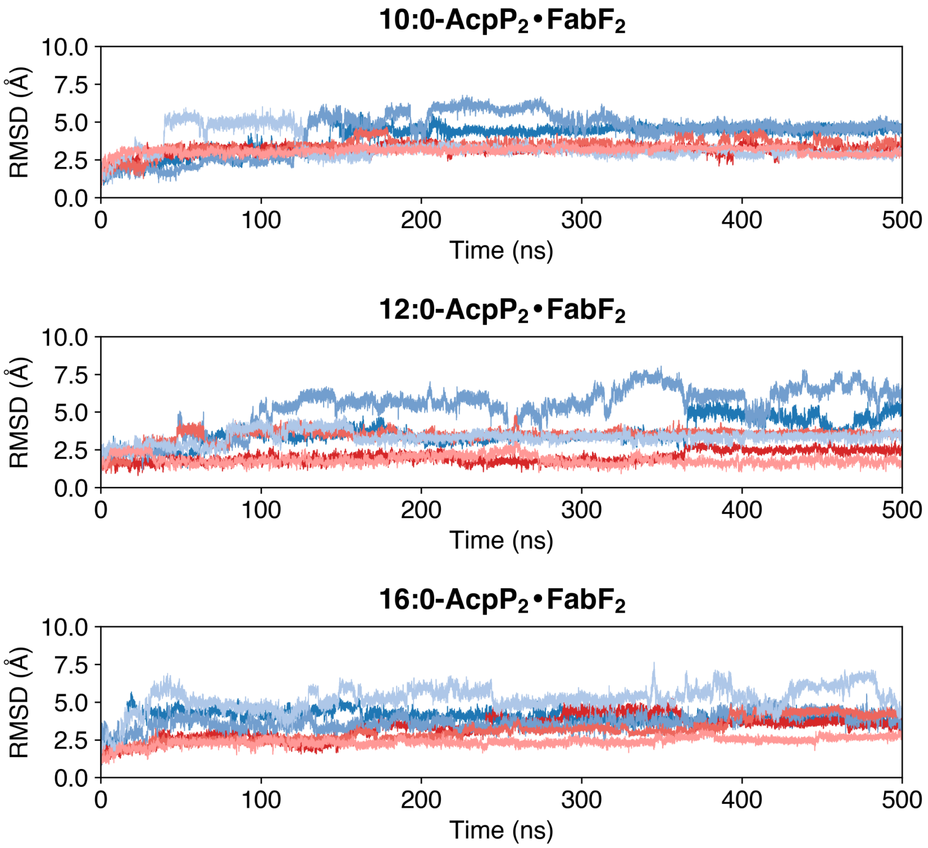

Supplementary Figure 27. RMSDs of the nonstandard residue of acyl-AcpP2•FabF2 complexes sampled during cMD simulations. Each blue (FabF1) and red (FabF2) curve represents data from an independent 500 ns simulation.

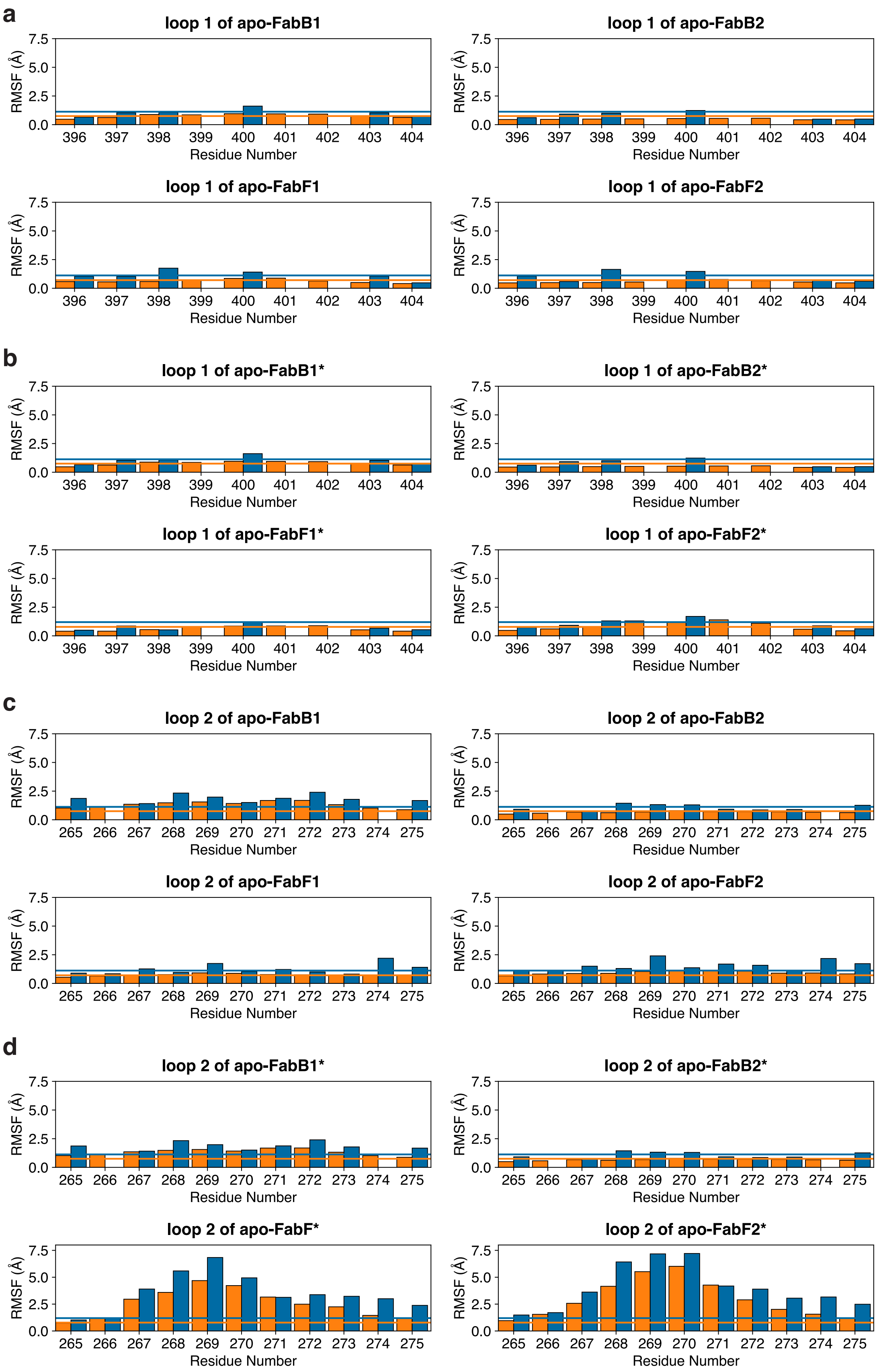
a
loop 1 of apo-FabB1
loop 1 of apo-FabB2
loop 1 of apo-FabF1
loop 1 of apo-FabF2
b
loop 1 of apo-FabB1*
loop 1 of apo-FabB2*
loop 1 of apo-FabF1*
loop 1 of apo-FabF2*
c
loop 2 of apo-FabB1
loop 2 of apo-FabB2
loop 2 of apo-FabF1
loop 2 of apo-FabF2
d
loop 2 of apo-FabB1*
loop 2 of apo-FabB2*
loop 2 of apo-FabF*
loop 2 of apo-FabF2*
RMSF (Å)
Residue Number
0.0
2.5
5.0
7.5
396 397 398 399 400 401 402 403 404
265 266 267 268 269 270 271 272 273 274 275

Supplementary Figure 28. Root mean square (RMS) fluctuations each residue of loops 1 and 2 of FabB and FabF monomers of the apo-AcpP•KS sampled during MD simulations. **a.** Per residue RMSF of loop 1 of either monomer of *apo*-KS dimer. **b.** Per residue RMSF of loop 1 of either monomer of *apo*-KS dimer derived from the crosslinked AcpP=KS (*apo*-KS*) structure. **c.** Per residue RMSF of loop 2 of either monomer of *apo*-KS dimer. **d.** Per residue RMSF of loop 2 of either monomer of apo-*KS* dimer derived from the crosslinked AcpP=KS (*apo*-KS*) structure. The bar graphs show the per residue backbone RMS fluctuations (blue, measured using backbone heavy atoms) and the side chain RMS fluctuation of sidechains (orange, measured using each residue's sidechain heavy atoms). Residues 396-404 and residues 265-275 (FabF residue numbering) define loops 1 and 2, respectively.

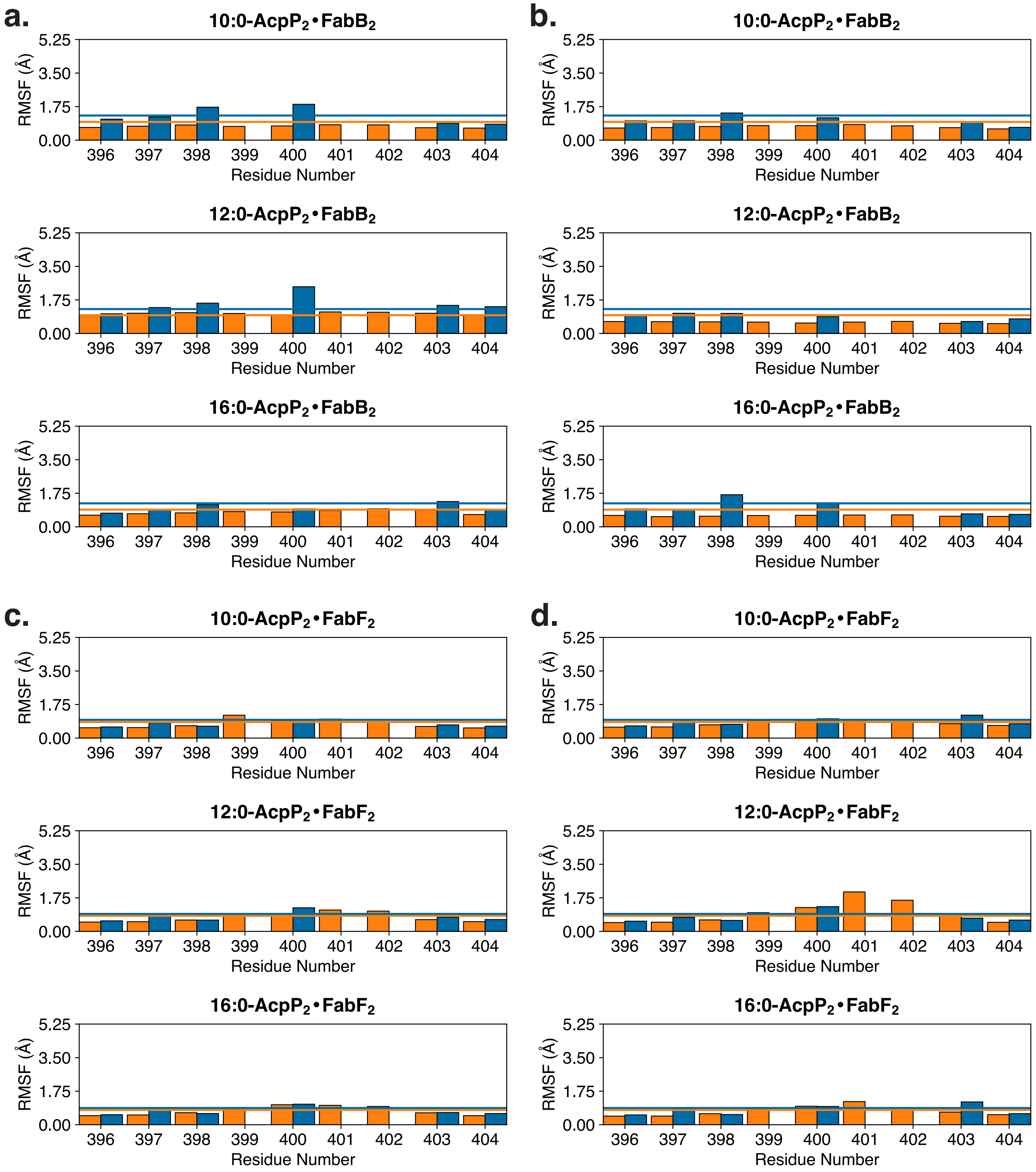

Supplementary Figure 29. Root mean square (RMS) fluctuations each residue of loop 1 of FabB and FabF monomers of the acyl-AcpP•KS sampled during MD simulations. a. Per residue RMSF of loop 1 of the FabB1 monomer of acyl-AcpP•FabB. **b.** Per residue RMSF of loop 1 of the FabB2 monomer of acyl-AcpP•FabB. **c.** Per residue RMSF of loop 1 of the FabF1 monomer of acyl-AcpP•FabF. **d.** Per residue RMSF of loop 1 of the FabF2 monomer of acyl-AcpP•FabF. The bar graphs show the per residue backbone RMS fluctuations (blue, measured using backbone heavy atoms) and the side chain RMS fluctuation of sidechains (orange, measured using each residue's sidechain heavy atoms). Residues 396-404 (FabF residue numbering) define loop 1.

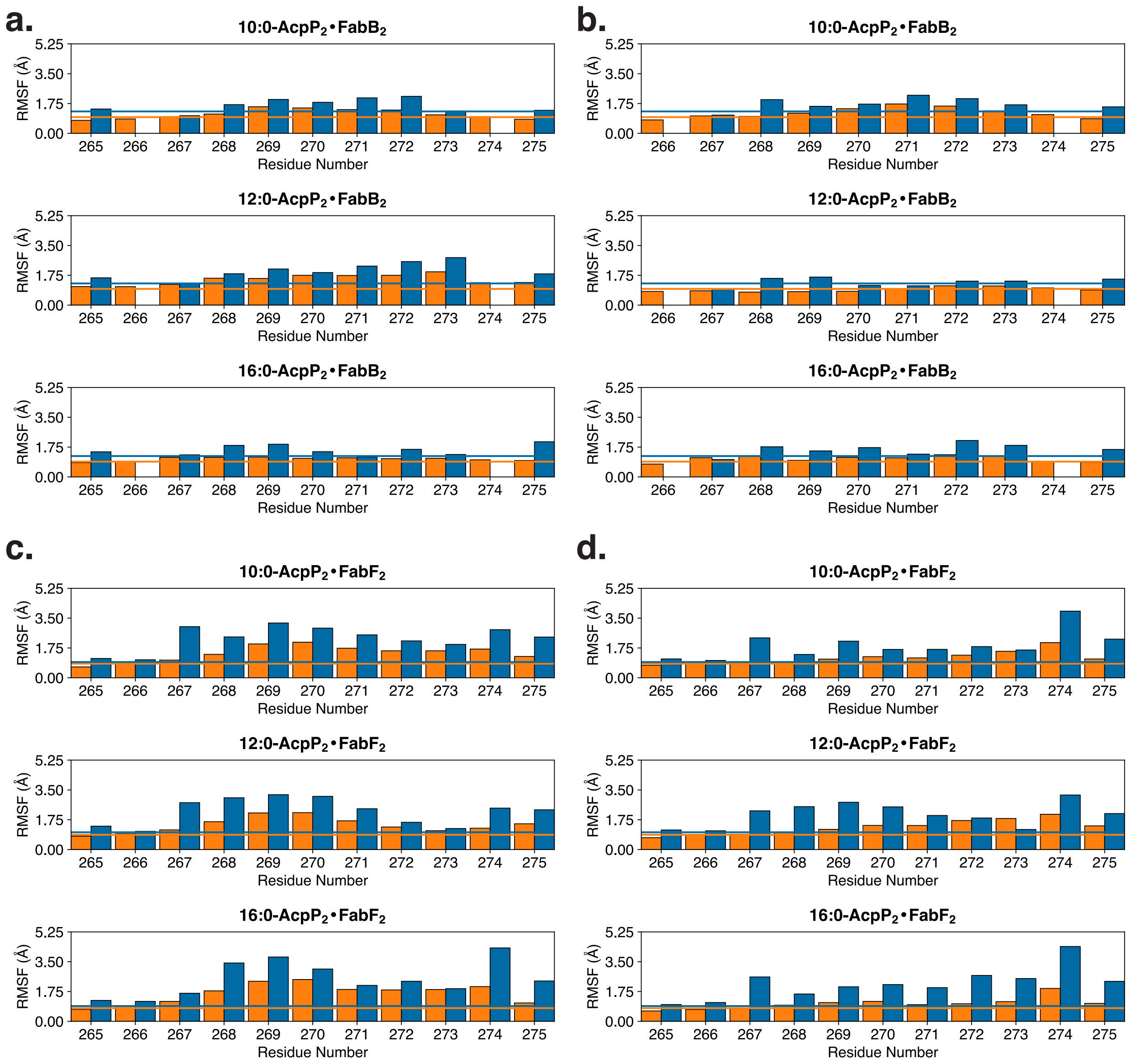

Supplementary Figure 30. Root mean square (RMS) fluctuations each residue of loop 2 of FabB and FabF monomers of the acyl-AcpP2•KS2 sampled during MD simulations. a. Per residue RMSF of loop 2 of the FabB1 monomer of acyl-AcpP•FabB. **b.** Per residue RMSF of loop 2 of the FabB2 monomer of acyl-AcpP•FabB. **c.** Per residue RMSF of loop 2 of the FabF1 monomer of acyl-AcpP•FabF. **d.** Per residue RMSF of loop 2 of the FabF2 monomer of acyl-AcpP•FabF. The bar graphs show the per residue backbone RMS fluctuations (blue, measured using backbone heavy atoms) and the side chain RMS fluctuation of sidechains (orange, measured using each residue's sidechain heavy atoms). Residues 265-275 (FabF residue numbering) define loop 2.

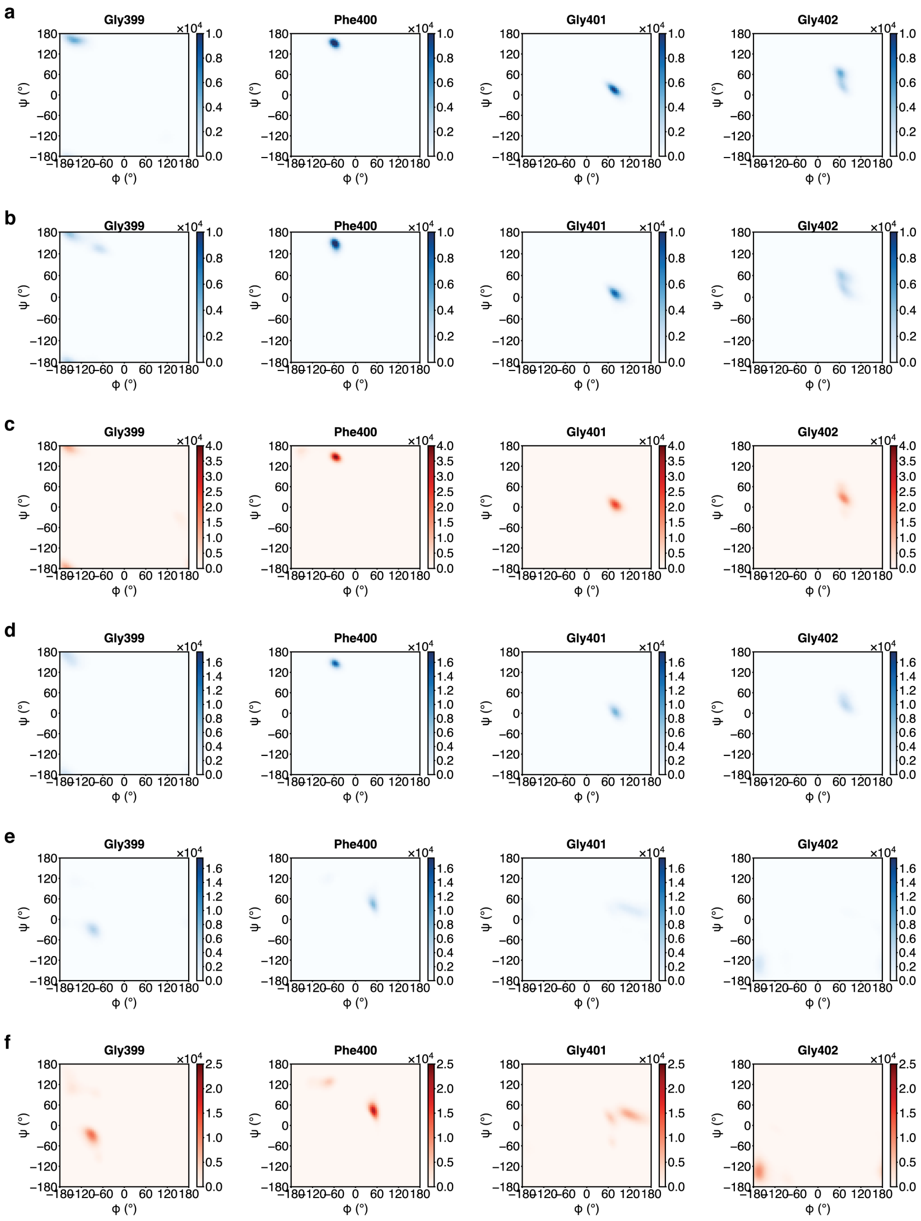

Supplementary Figure 31. Ramachandran plots of the GFGG motifs of FabB or FabF simulated in various states. a. Analysis of the GFGG backbone dihedrals of *apo*-FabB performed using 1.5 µs of molecular dynamics (MD) simulation data of *apo*-FabB. **b.** Analysis of GFGG backbone dihedrals on apo-FabB* (apo-FabB constructed using the coordinates from the crosslinked AcpP=FabB structure reported herein) using 1.5 µs of molecular dynamics (MD) simulation data of *apo*-FabB*. **c.** Analysis of the GFGG backbone dihedral of acyl-AcpP•FabB using 4.5 µs of molecular dynamics (MD) simulation data of acyl-AcpP•FabB. **d.** Analysis of the GFGG backbone dihedrals of *apo*-FabF performed using 1.5 µs of molecular dynamics (MD) simulation data of *apo*-FabF. **e.** Analysis of GFGG backbone dihedrals on apo-FabF* (apo-FabF constructed using the coordinates from the crosslinked AcpP=FabF structure reported herein) using 1.5 µs of molecular dynamics (MD) simulation data of *apo*-FabF*. **f.** Analysis of the GFGG backbone dihedral of acyl-AcpP•FabF using 4.5 µs of molecular dynamics (MD) simulation data of acyl-AcpP•FabF. Data is binned based backbone ψ and ϕ dihedral angles residues assume during MD simulations using bin widths of 5°

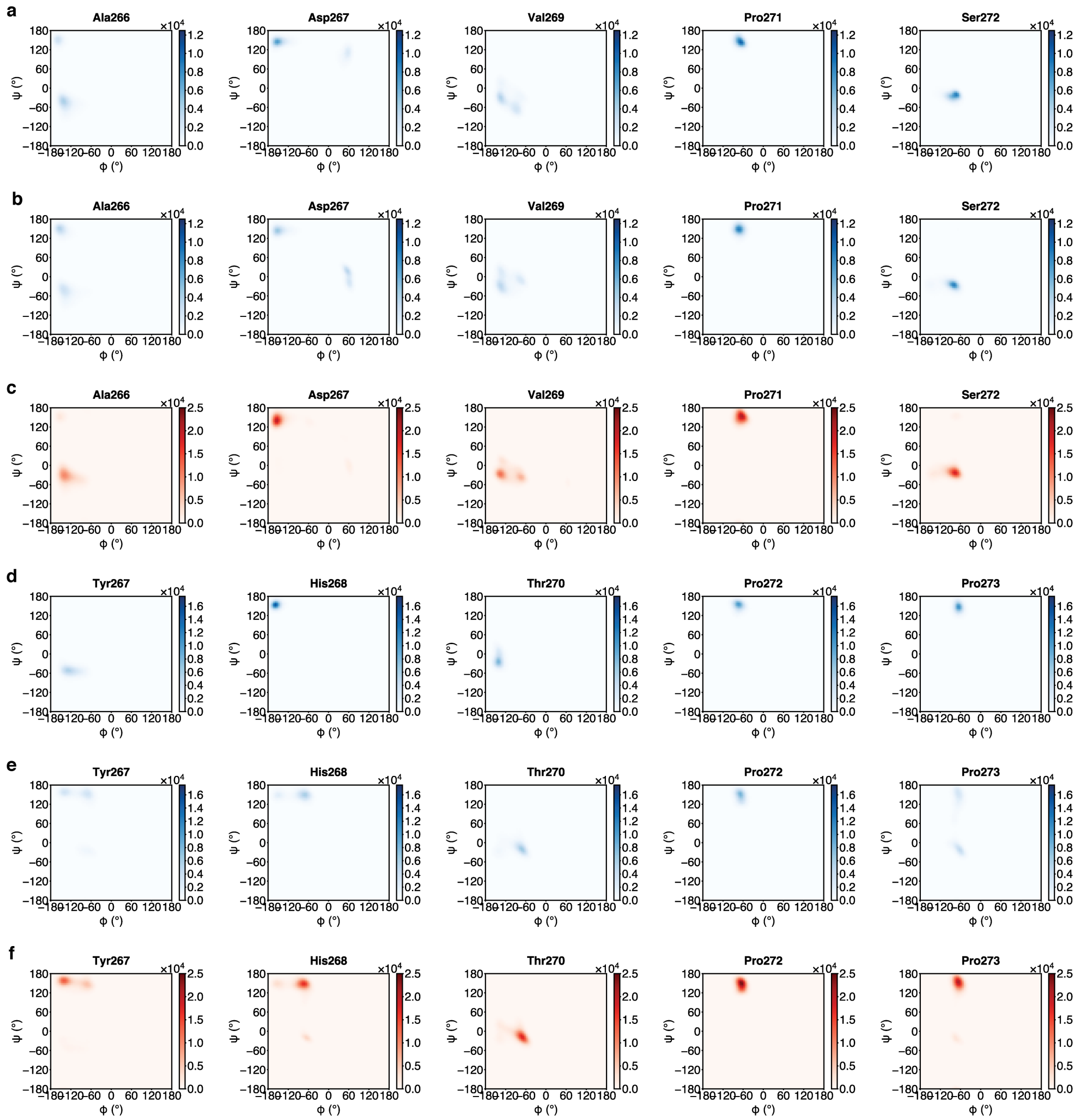

Supplementary Figure 32. Ramachandran plots of the key residues of loop 2 of FabB or FabF simulated in various states. a. Analysis of the loop 2 backbone dihedrals of *apo*-FabB performed using 1.5 µs of molecular dynamics (MD) simulation data of *apo*-FabB. **b.** Analysis of loop 2 backbone dihedrals on apo-FabB* (apo-FabB constructed using the coordinates from the crosslinked AcpP=FabB structure reported herein) using 1.5 µs of molecular dynamics (MD) simulation data of *apo*-FabB*. **c.** Analysis of the loop 2 backbone dihedrals of acyl-AcpP•FabB using 4.5 µs of molecular dynamics (MD) simulation data of acyl-AcpP•FabB. **d.** Analysis of the loop 2 backbone dihedrals of *apo*-FabF performed using 1.5 µs of molecular dynamics (MD) simulation data of *apo*-FabB. **e.** Analysis of loop 2 backbone dihedrals on apo-FabF* (apo-FabF constructed using the coordinates from the crosslinked AcpP=FabF structure reported herein) using 1.5 µs of molecular dynamics (MD) simulation data of *apo*-FabB*. **f.** Analysis of the loop 2 backbone dihedral of acyl-AcpP•FabF using 4.5 µs of molecular dynamics (MD) simulation data of acyl-AcpP•FabF. Data is binned based backbone ψ and φ dihedral angles residues assume during MD simulations using bin widths of 5°.

Primer	Sequence
FabF-G399A-F	GCGTTCGGTGGCACTAATGGTTCTTTGATCTTTAAAAAGATCTAA
FabF-G399A-R	AGTGCCACCGAACGCGAAGGAGTTACACAGAGTGTATTC
FabF-F400A-F	*GGCGCAGGTGGCACTAATGGTTCTTTGATCTTTAAAAAGATCTAA*
FabF-F400A-R	AGTGCCACCTGCGCCGAAGGAGTTACACAGAGTGTATTC
FabF-G401A-F	*GGCTTCGCGGGCACTAATGGTTCTTTGATCTTTAAAAAGATCTAA*
FabF-G401A-R	AGTGCCCGCGAAGCCGAAGGAGTTACACAGAGTGTATTC
FabF-G402A-F	*GGCTTCGGTGCGACTAATGGTTCTTTGATCTTTAAAAAGATCTAA*
FabF-G402A-R	AGTCGCACCGAAGCCGAAGGAGTTACACAGAGTGTATTC
FabF-D265A-F	*ATG AGC AGC GCA GCT TAT CAT ATG ACG TCA CCG CCA GAA AAT G*
FabF-D265A-R	ATA AGC TGC GCT GCT CAT ACC AAA GCC GAC GAG TTC AGC
FabF-D265N-F	*ATG AGC AGC AAC GCT TAT CAT ATG ACG TCA CCG CCA GAA AAT G*
FabF-D265N-R	ATA AGC GTT GCT GCT CAT ACC AAA GCC GAC GAG TTC AGC
FabF-G310F-F	*TCT ACG CCG GCT TTT GAT AAA GCT GAA GCG CAG GCG GTG*
FabF-G310F-R	ATC AAA AGC CGG CGT AGA AGT ACC GTG CGC GTT AAC GTA
FabF-G310M-F	*TCT ACG CCG GCT ATG GAT AAA GCT GAA GCG CAG GCG GTG*
FabF-G310M-R	ATC CAT AGC CGG CGT AGA AGT ACC GTG CGC GTT AAC GTA

Supplementary Figure 33. Primers for generating FabF mutants. F and R indicate forward and reverse primers

Supplementary Figure 34. Table 1 Refinement statistics from structures solved in this work.

	C16AcpP-FabB	C12AcpP-FabB	C16AcpP-FabF	C12AcpP-FabF
**Data collection**				
Space group	P 1 21 1	P 21 21 21	P 43 21 2	P 43 21 2
Cell dimensions				
a, *b*, *c* (Å)	58.94 100.78 79.26	59.07 112.17 140.68	86.89 86.89 113.73	86.45 86.45 115.16
α, β, γ (°)	90.00 108.64 90.00	90.0 90.0 90.0	90.0 90.0 90.0	90.0 90.0 90.0
Resolution (Å)	60.22-2.5(2.59-2.5)*	87.7-1.55(1.60-1.55)*	69.04-2.3(2.382-2.3)*	69.14-2.35(2.43-2.35)*
R_{sym} or R_{merge}	0.136 (.8075)	0.109(0.857)	0.3746 (2.379)	0.327 (3.604)
$I / \sigma I$	7.59 (2.34)	14.25 (3.65)	9.28 (1.70)	9.91 (1.08)
Completeness (%)	98.74 (98.19)	99.43 (98.28)	99.80 (99.95)	99.74 (99.67)
Redundancy	6.2 (6.4)	11.8 (11.7)	19.1 (19.9)	15.4 (15.9)
**Refinement**				
Resolution (Å)	60.22-2.5	87.7-1.55	69.04-2.3	69.14-2.35
No. reflections	300780 (2991)	1593391 (155305)	381910 (38754)	288893 (29114)
R_{work} / R_{free}	.178/.218	.148/.163	.195/.228	.201/.239
No. atoms	7337	8287	3914	3867
Protein	7160	7343	3640	3727
Ligand/ion	70	102	77	48
Water	107	842	197	92
B-factors	65.50	22.28	31.90	59.21
Protein	66.25	20.97	31.87	59.54
Ligand/ion	61.03	28.79	33.43	58.36
Water	50.24	32.86	31.91	46.56
R.m.s. deviations				
Bond lengths (Å)	.004	.008	.004	.004
Bond angles (°)	.99	1.3	.97	.96

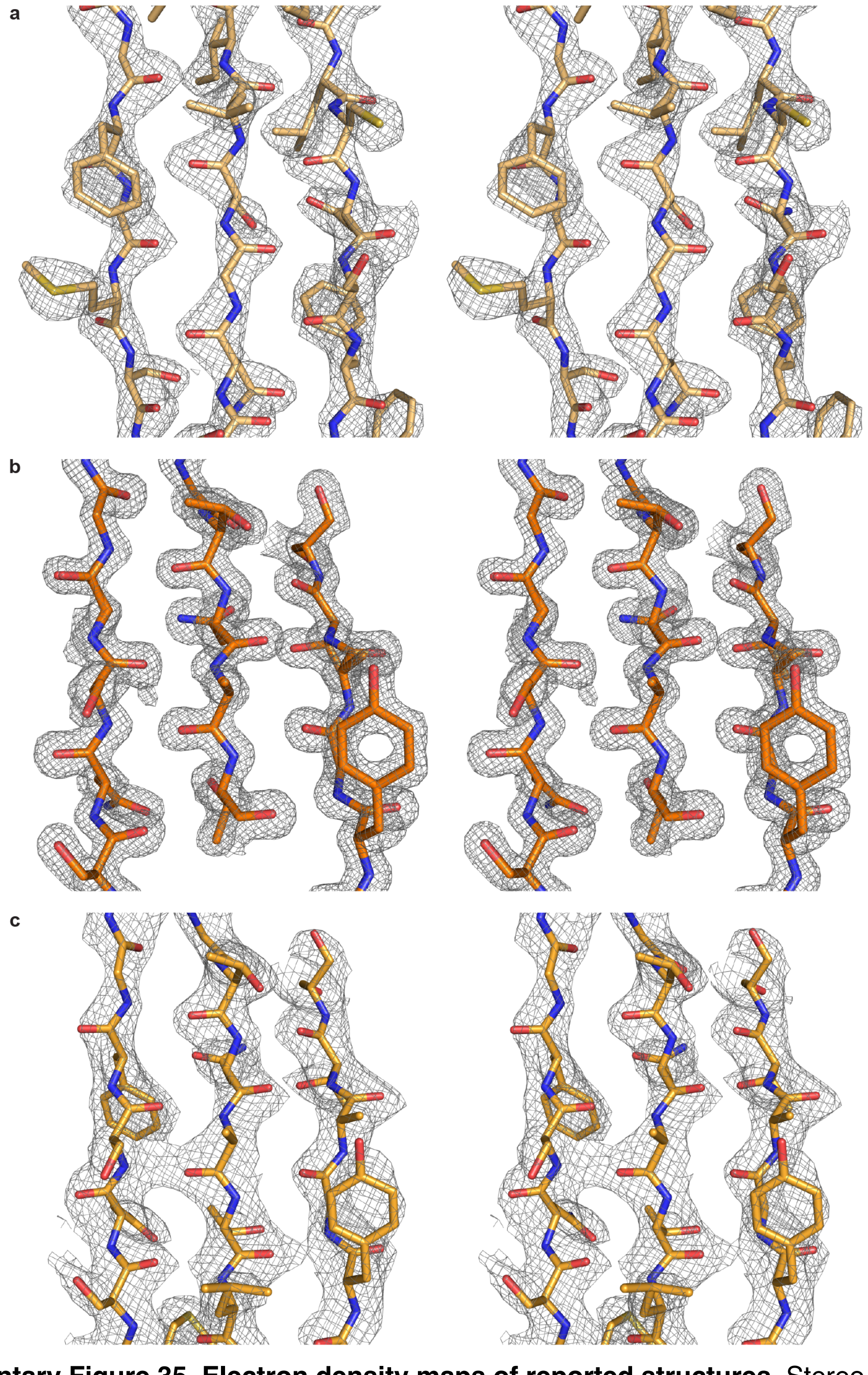

Supplementary Figure 35. Electron density maps of reported structures. Stereo images of 2Fo-Fc electron density maps contoured at 1.5 σ for **a.** C16AcpP-FabF, (PDB: 6OKG, 2.4 Å), beta sheet shown consists of residues Leu257-Ser263, Thr393-G399, and Thr403-Phe409. b. C12AcpP-FabB, (PDB: 6OKC ,1.55 Å), beta sheet shown consists of residues Gly259-Ser264, Gly394-Thr398, and Ser387-F392. c. C16AcpP-FabB, beta sheet shown consists of residues Gly259-Ser264, Gly394-Thr398, and Ser387-F392 (PDB: 6OKF, 2.5 Å.)

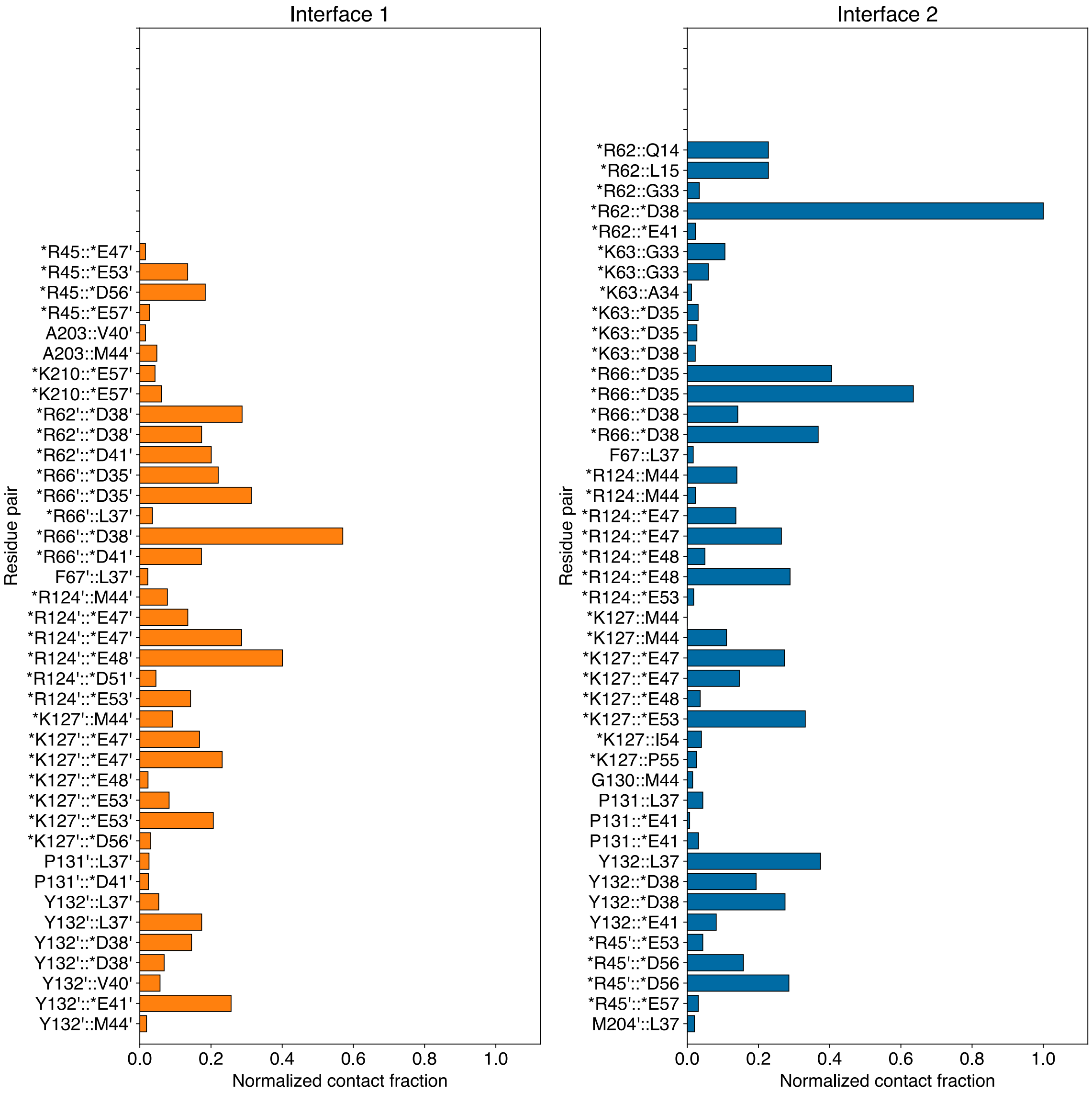

Supplementary Figure 36. Native contact analysis of MD simulations data of acyl-AcpP2•FabB2 (10:0-AcpP2•FabB2, 12:0-AcpP2•FabB2, 16:0-AcpP2•FabB2). Residues on the dimeric $FabB_2$ subunit within 7.5 Å and those of either AcpP1 (Interface 1) and AcpP2 (Interface 2) within 7.5 Å dimer subunit were subjected to this analysis. The bar graphs illustrate the frequency with interfacial residues contact with one another at either interface of these acyl-$AcpP_2$•$FabB_2$. The contact fraction is defined as the total fraction of simulation data in which a residue pair is engaged in an intermolecular contact. A distance criterion of 3.5 Å or less between a pair of heavy atoms of two residue belonging two distinct protein monomers defines such a contact. This value before normalization assume values greater than 1.0, if a residue pair possesses multiple atoms that satisfy the distance criteria used to define a contact. Only pairwise contacts with contact fractions greater than or equal to 0.05 are included in the plots above. Every 10th frame of simulation data starting with the first was used to perform this analysis. Note that “primed” residues (e.g., K127’, D35’) refer to residues belonging to FabB2 or AcpP2. Charged residues are marked by an asterisk.

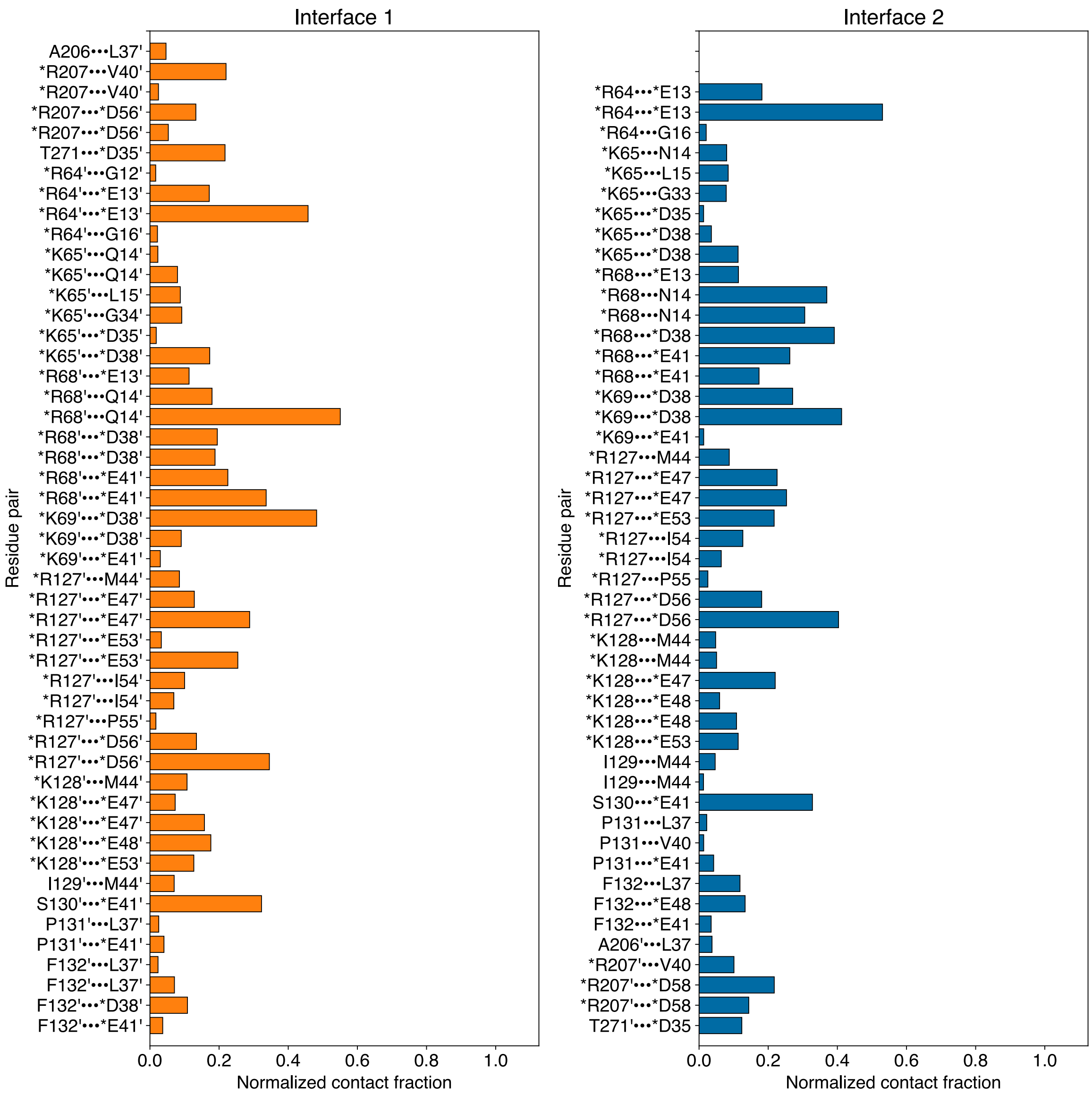

Supplementary Figure 37. Native contact analysis of MD simulations data of acyl-AcpP2•FabF2 (10:0-AcpP2•FabF2, 12:0-AcpP2•FabF2, 16:0-AcpP2•FabF2). Residues on the dimeric $FabF_2$ subunit within 7.5 Å and those of either AcpP1 (Interface 1) and AcpP2 (Interface 2) within 7.5 Å dimer subunit were subjected to this analysis. The bar graphs illustrate the frequency with interfacial residues contact with one another at either interface of these acyl-$AcpP_2$•$FabF_2$. The contact fraction is defined as the total fraction of simulation data in which a residue pair is engaged in an intermolecular contact. A distance criterion of 3.5 Å or less between a pair of heavy atoms of two residue belonging two distinct protein monomers defines such a contact. This value before normalization assume values greater than 1.0, if a residue pair possesses multiple atoms that satisfy the distance criteria used to define a contact. Only pairwise contacts with contact fractions greater than or equal to 0.05 are included in the plots above. Every 10th frame of simulation data starting with the first was used to perform this analysis. Note that “primed” residues (e.g., K127’, D35’) refer to residues belonging to FabF2 or AcpP2. . Charged residues are marked by an asterisk.

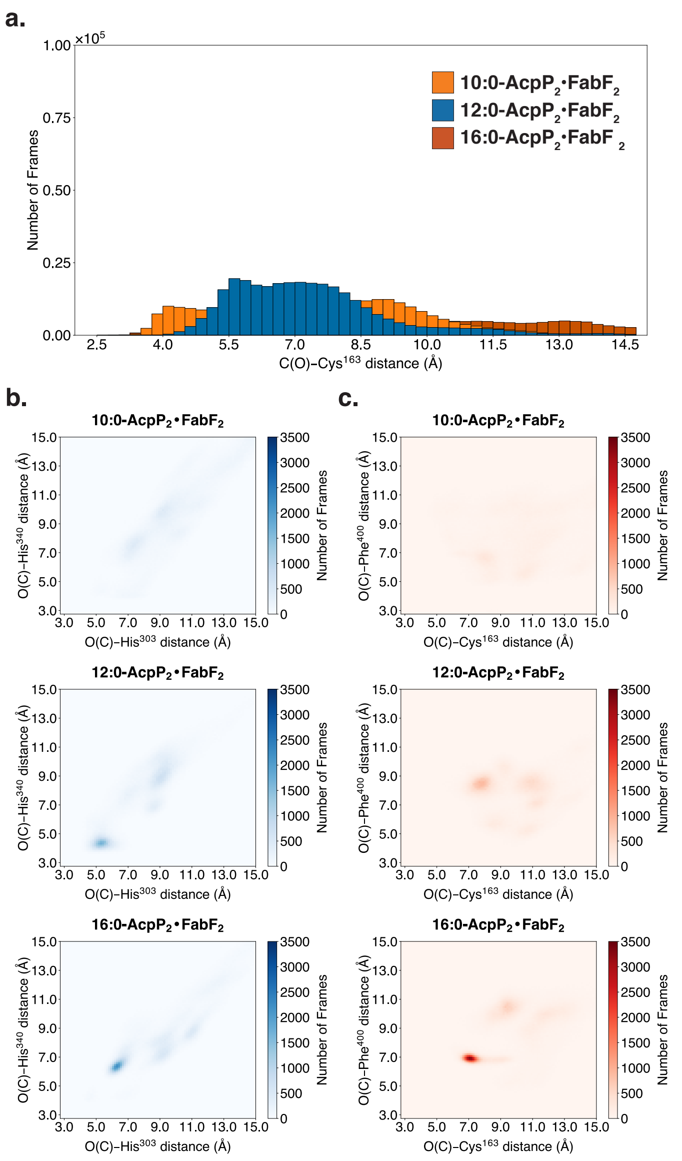

Supplementary Figure 38. Analysis of contacts between active site residues and substrate of acyl-AcpP•FabF complexes sampled computationally. a. Distribution of distances sampled between carbonyl carbon of the acyl chain of 10:0-AcpP•FabF (orange), 12:0-AcpP-FabF (blue), or 16:0-AcpP•FabF (red) and sulfur atom of catalytic cysteine (Cys163). 1D histogram was generated using a bin width of 0.25 Å **b.** Analysis of hydrogen bonding interactions involving the substrate and His303 and His340 (2D histogram, blue). Simulation data is binned along two coordinates The first of which is the distance between the carbonyl oxygen of the substrate and the imidazole moiety of His303 (center of geometry) of FabF, while the second measures the distance between the imidazole moiety of His340 (center of geometry) and its carbonyl oxygen. **c.** Analysis of backbone (oxyanion hole) hydrogen-bonding interactions (2D histogram, red). Simulation data is binned along two coordinates. The first of which is the distance between the carbonyl oxygen of the substrate and the backbone amide nitrogen of Cys163 of FabF, while the second measures the distance between substrate's carbonyl oxygen and the backbone amide nitrogen of Phe400. Simulation data is binned along two coordinates. Data was sorted into 0.05 Å × 0.05 Å bins. Color bars indicate the absolute population of each histogram bin.

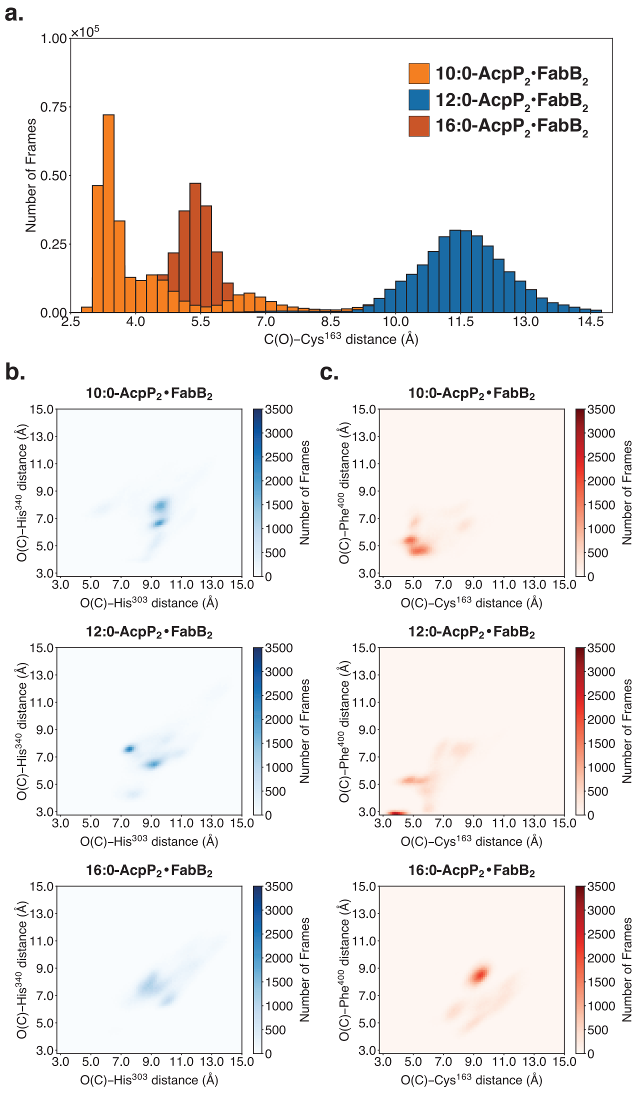

Supplementary Figure 39. Analysis of contacts between active site residues and substrate of acyl-AcpP•FabB complexes sampled computationally. a. Distribution of distances sampled between carbonyl carbon of the acyl chain of 10:0-AcpP•FabB (orange), 12:0-AcpP-FabB (blue), or 16:0-AcpP•FabB (red) and sulfur atom of catalytic cysteine (Cys163). 1D histogram was generated using a bin width of 0.25 Å **b.** Analysis of hydrogen bonding interactions involving the substrate and His303 and His340 (2D histogram, blue). Simulation data is binned along two coordinates The first of which is the distance between the carbonyl oxygen of the substrate and the imidazole moiety of His303 (center of geometry) of FabB, while the second measures the distance between the imidazole moiety of His340 (center of geometry) and its carbonyl oxygen. **c.** Analysis of backbone (oxyanion hole) hydrogen-bonding interactions (2D histogram, red) Simulation data is binned along two coordinates. The first of which is the distance between the carbonyl oxygen of the substrate and the backbone amide nitrogen of Cys163 of FabB, while the second measures the distance between substrate's carbonyl oxygen and the backbone amide nitrogen of Phe400. Simulation data is binned along two coordinates. Data was sorted into 0.05 Å × 0.05 Å bins. Color bars indicate the absolute population of each histogram bin.

Supplementary Note 1. C12AcpP-FabF complex

The C12AcpP-FabF complex crystalized in the same conditions and space group as our C16AcpP-FabF structure and produced electron density maps of similar quality (Supplementary Fig. 34). Structural alignments of the two complexes show that C12AcpP-FabF overlays with C16AcpP-FabF with an RMSD of 0.19 Å. The electron density map is well defined for the majority of the complex except for loops 1 and 2, which appear to be dynamic in the C12AcpP-FabF structure. These loops adopt at least two conformations, which are representative of apo-FabF (closed state) and our C16 bound state (open state), making the interpretation of these regions difficult. Given the presence of electron density for both the open and closed states, both loop conformations were modelled for loops 1 and 2.

Analysis of the PPant crosslinking probe demonstrates discernable density for the PPant arm but not for the C12 alkyl chain. This is not surprising as the conformational heterogeneity in loops 1 and 2 make it difficult to accurately model this region of the complex. Therefore, the finalized structure does not include an alkyl chain, which is instead modelled as a C2 with a crosslink at the two-position (α position). The lack of density for the alkyl chain may indicate that it is dynamic and that the gate is switching between the open and closed conformation. Extension of the chain four carbons from C12 to C16, as seen in our C16AcpP-FabF structure, appears to stabilize the open over the closed conformation, which is likely due to the large size of the alkyl chain.

Supplementary Note 2. C16AcpP-FabB complex

The 2.50 Å C16AcpP-FabB x-ray refinement statistics and electron density map can be found in Supplementary Fig. 34,35c. The C16AcpP-FabB structure overlays with the C12AcpP-FabB with a root-mean-square deviation (RMSD) of 0.25 Å. Despite the overall structural similarities, the C16AcpP-FabB data reveals a lack of electron density for the C16-acyl chain in the acyl-binding pocket, despite showing interpretable electron density for the 4'-phosphopantetheineamide portion of the probe (Supplementary Fig. 4f). A C12-acyl chain was modeled into the acyl-binding pocket, but low levels of electron density were only observed for the alkyl chain in 2Fo-Fc maps at or below contour levels of 1σ (Supplementary Fig. 4f). A similar result was found by Olsen et al.[42] in a study where they soaked acyl-CoAs into FabB crystals to trap the FabB acyl-enzyme adduct with different fatty acid chain lengths.[1] Their soaking experiments only showed strong density for C8, C10, and C12 chains but little to no electron density with C14 and C16. Despite the lack of alkyl chain density, we attempted to model the complete C16 alkyl chain into the FabB binding pocket, but in doing so recognized a constricted fatty acid binding pocket that places the alkyl chains of the two protomers in close proximity to one another at the dimer interface (Supplementary Fig. 4g). Furthermore, clashes between the terminal carbons of the C16-acyl chain with Glu200 and Gln113 along the back wall of the binding pocket restrict accommodation of the C16 substrate. Interestingly, in our C12AcpP-FabB datasets, the end of the C12 chain stops just short of these residues (Supplementary Fig. 4e), indicating that they may play a role in the substrate specificity of FabB.[2–5]

Supplementary Note 3. Time resolved analysis of AcpP-KS interfaces

MD simulations of the various acyl-AcpP-KS complexes (Supplementary Fig. 13) show the extent to which atoms interact intermolecularly at the AcpP-KS interface. FabF, unlike FabB, forms long-lived contacts with AcpP's helix I residues Glu13 and Gln14 during the course of the simulations (Fig. 2, Supplementary Fig. 36,37). FabF forms additional short-lived hydrophobic contacts with AcpP throughout the MD simulations, consistent with the larger contact area of the AcpP-FabF interface (Supplementary Fig. 36,37). Simulations also reveal that the charged residues of regions 1 and 2 of the AcpP-KS interface form and break interactions with one another throughout the course of the simulation (Supplementary Fig. 36,37), showcasing the dynamism of the PPIs of these complexes. Simulations show that AcpP's Asp35 and Asp38 are the residues most frequently forming interactions at the AcpP-FabB interface (Supplementary Fig. 36,37).

Supplementary Note 4. C16AcpP-FabF delineates a potential minor conformation for the C16 alky chain

The refined structure of C16AcpP-FabF may delineate density for a second conformation of the alkyl chain, which we define as C16b, that passes towards the dimer interface in the opposite direction of the fatty acyl-binding pocket (Supplementary Fig. 4a-c). Movement of loop 1 to the open conformation creates a hydrophobic cavity of significant size with in the FabF active site, potentially allowing access to this minor conformation by a rotation around the C5-C6 bond of the alkyl chain. Modeling a C16 chain into this density is possible and the chain is not constricted by the size of the pocket. A comparison of the C16a and C16b conformations shows significantly higher electron density for the C16a conformation, but both conformations have been modeled into the final structure. Analysis of the substrate-protein interactions show that the C16a conformation has significantly more favorably hydrophobic contacts than that of the C16b pocket (Supplementary Fig. 4g). The composition of the pocket surrounding the alkyl chain in the C16b conformation is slightly more polar than that of the C16a conformation and is comprised of residues Ile138, Asn140, Met141, Thr161, Ala162, Met269, and Thr403 (Supplementary Fig. 4g).

Supplementary Note 5. Comparison of FabF and FabB structural features

E. coli and most other γ-proteobacteria possess three ketosynthases which are representatives of the KASI, KASII, and KASIII families of condensing enzymes.[6] KASIII enzymes are also considered initiating KSs and serve to perform the first condensation reaction of FAS by condensing acetyl-CoA with malonyl-AcpP. All subsequent extensions are performed by the elongating KSs, KASI and KASII. FabH is the KASIII representative in E. coli, while FabB and FabF are the KASI and KASII representatives, respectively. Early work by Vagelos, Cronan, and Rock established differences in activity and substrate specificity for the *E. coli* elongating KSs, FabB and FabF.[2–4,7] *In vivo*, FabB, in conjunction with the dehydratase FabA, serve to create a branchpoint responsible for the production of unsaturated fatty acids from *de novo* FAB.[7–10] FabA isomerizes the *trans*-2-decenoyl-AcpP intermediate to *cis*-3-decenoyl-AcpP and FabB preferentially elongates *cis*-3-decenoyl-AcpP to *cis*-5-dodecenoyl-AcpP. The condensation reaction catalyzed by FabB at the branchpoint is essential as the FabA catalyzed isomerization is reversible. Therefore, FabB extension of *cis*-3-decenoyl-AcpP immortalizes the unsaturation, thereby producing the unsaturated fatty acids branch of the FAB pathway in *E. coli*. Additionally, Cronan and coworkers demonstrated that FabF regulates membrane fluidity in response to changes in temperature through the conversion of *cis*-palmitoleoyl-AcpP (16:1-AcpP) into *cis*-vaccenoyl-AcpP (18:1-AcpP) at lower temperatures *in vivo* and *in vitro*, thereby increasing the amount of unsaturated fatty acids present in the membrane.[4,11]

Given the differences in reactions catalyzed by FabB and FabF, it is reasonable to assume that there are some important structural differences in these two enzymes. Indeed, x-ray structures of these synthases have shown that there are additional gating residues that control access to the fatty acid binding pocket. In FabF, this gate is composed of Ile108 and F202 while in FabB Ile108 is replaced by a glycine residue. Replacement of the bulky Isoleucine for a small

flexible glycine likely allows more facile access to the FabB substrate binding pocket. Additional differences between these synthases can also be found in their fatty acid binding pockets. The FabF acyl-binding pocket appears more hydrophobic than that of FabB, which is somewhat truncated at its back wall by Gln113 and Glu200. These two residues come together to form a hydrogen bond that decreases the depth of this pocket (Supplementary Fig. 4e). Interestingly, there appears to be crosstalk between the two FabB protomers mediated by these two residues. Gln113 sits at the dimer interface and is present in two conformations in our C12AcpP-FabB structure, one of which shortens the binding pocket and the other where Gln113 can move away from Glu200 and provide more space for an alkyl chain. Movement of Gln113 away from Glu200 can only happen in one binding pocket at a time as the Gln113 from the neighboring protomer would occupy the same location. Effectively, a small pocket exists at the interface of the two protomers and only one Gln113 can occupy this space at a time, potentially providing a mechanism for one protomer to affect the substrates specificity of the other.

Despite these structural differences, the molecular mechanisms responsible for the distinct activities of FabF and FabB remain unknown. The proposal of a double drawbridge gating system coordinated by loops 1 and 2 may provides a new approach to answering some of these questions. Gates can be evolutionarily tuned for certain substrates or a certain activity[12], and it may be that differences in the loop 2 sequence of FabF and FabB may be a good place to start. This may still be complicated by the previously listed differences, namely the additional gate regulating access to the acyl-binding pocket. FabB lacks the Ile108 present in FabF, and it may be that differences in both gates play a role in determining substrate specificity and may be effectively tuned to one another to favor or disfavor entry of certain substrates. Moving forward, a holistic approach looking at multiple aspects of the actives sites of FabF and FabB will likely be needed to answer some of the remaining questions for these enzymes.

Some of the differences in structure and sequence between FabF and FabB may also be responsible for stabilizing the conformations seen in our trapped AcpP-KS complexes. Both FabF structures solved in this work demonstrate a gate open conformation, although in the case of C12AcpP-FabF both the open and closed conformations appear to be present. On the other hand, both C12AcpP-FabB and C16AcpP-FabB complexes present a gate closed conformation with the alkyl chain placed into the acyl-binding pocket. The easiest explanation for such a result might be that FabB has a single gating residue (Phe201) regulating access to the acyl-binding pocket while FabF has two (Ile108 and Phe202). In FabF, Ile108 has to rotate away from Phe202 to create space for the alkyl chain in the dodecanoyl bound FabF structure, 2GFY[13]. With the bound AcpP present, it may be that the AcpP-FabF complex favors a gate open conformation while FabB favors a gate closed conformation because it can more easily accommodate the alkyl chain in the acyl-binding pocket. Another difference in these complexes is their loop 2 sequences, which are not conserved between the KASI and KASII condensing enzyme families. The fact that Thr270 of FabF interacts with Asp35 of the bound AcpP molecule is an interesting point and it may be that the bound AcpP molecule helps stabilize the gate open conformation, as proposed in the main text. FabF's loop 2 is also stabilized by an interesting CH-π interaction found between Pro273 and Tyr267, which is not found in FabB. Additionally, these differences, together, may also be responsible for the ability to trap these complexes in different conformations.

Supplementary Note 6. Substrate interaction analysis in AcpP•FabF simulations

Unlike C16AcpP-FabF, MD simulations suggest that the acyl chain carbonyls in 10:0-AcpP-FabF, 12:0-AcpP-FabF, 16:0-AcpP-FabF complexes do not form hydrogen bonds with either active site histidine (Fig. 3a). Over the course of the simulation, the substrate relaxes to assume a distinct (pre-transacylation/post-condensation) disposition within the hydrophobic pocket. Furthermore, the oxyanion hole comprising Cys163 and Phe400 backbone amides was not engaged by the substrate during the course of the simulations (Supplementary Fig. 38). These findings, in addition to long distances ($d > 5.0$ Å) between the substrates and the thiolate moiety of the active site cysteine (Supplementary Fig. 38), suggest the AcpP-FabF structure features a pose wherein the crosslinker mimics a substrate conformation prior to transacylation, or a production conformation before dissociation.

Supplementary Note 7. Substrate interaction analysis in AcpP•FabB simulations

MD simulations of the acyl-AcpP-FabB complexes suggest that the dynamic features of these active sites are consistent with an active site preorganized for transacylation. The active site cysteine's thiolate moiety is significantly closer to the electrophilic carbon of the substrate (ca. 3.0 Å < d < 5.5 Å) than is observed in MD simulations of acyl-AcpP-FabF complexes (Supplementary Fig. 39). Backbone hydrogen-bonding interactions involving the substrate, Cys163, and Phe400 were only maintained in simulations of 12:0-AcpP-FabB (Supplementary Fig. 39). These interactions are disrupted during simulations of 16:0-AcpP-FabB, whereas in simulations of 10:0-AcpP-FabB, the substrate is ~5 Å away from either backbone hydrogen-bonding donor. Finally, no hydrogen bonds between the active site histidines (His303 and His340) and any substrate modeled are sampled during MD simulations (Supplementary Fig. 39).

Supplementary Note 8. General synthetic methods. Chemical reagents were purchased from Acros, Fluka, Sigma-Aldrich, or TCI. Deuterated NMR solvents were purchased from Cambridge Isotope Laboratories. All reactions were conducted with vigorously dried anhydrous solvents that were obtained by passing through a solvent column exposed of activated A2 alumina. All reactions were performed under positive pressure of argon in flame-dried glassware sealed with septa, and stirring with Teflon coated stir bars using an IKAMAG TCT-basic mechanical stirrer (IKA GmbH). Analytical Thin Layer Chromatograpy (TLC) was performed on Silica Gel 60 F254 precoated glass plates (EM Sciences). Visualization was achieved with UV light and/or appropriate stain (I_2 on SiO_2, $KMnO_4$, bromocresol green, dinitrophenylhydrazine, ninhydrin, or ceric ammonium molybdate). Flash column chromatography was carried out with Geduran Silica Gel 60 (40–63 mesh) from EM Biosciences. Yield and characterization data correspond to isolated, chromatographically, and spectroscopically homogeneous materials. ^{1}H NMR spectra were recorded on Varian Mercury 400 or Varian Mercury Plus 400 spectrometers. ^{13}C NMR spectra were recorded at 100 MHz on Varian Mercury 400 or Varian Mercury Plus 400 spectrometers. Chemical shifts for ^{1}H NMR and ^{13}C NMR analyses were referenced to the reported values of Gottlieb[14] using the signal from the residual solvent for ^{1}H spectra, or to the ^{13}C signal from the deuterated solvent. Chemical shift δ values for the ^{1}H and ^{13}C spectra are reported in parts per millions (ppm) relative to these referenced values, and multiplicities are abbreviated as s=singlet, d=doublet, t=triplet, q-quartet, m=multiplet, b=broad. All ^{13}C NMR spectra were recorded with complete proton decoupling. FID files were processed using MestreNova 10.0 (MestreLab Research).

Electrospray ionization (ESI) mass spectrometric analyses were preformed using a ThermoFinnigan LCQ Deca spectrometer. Spectral data, procedures, and copies of spectra are provided for all new compounds.

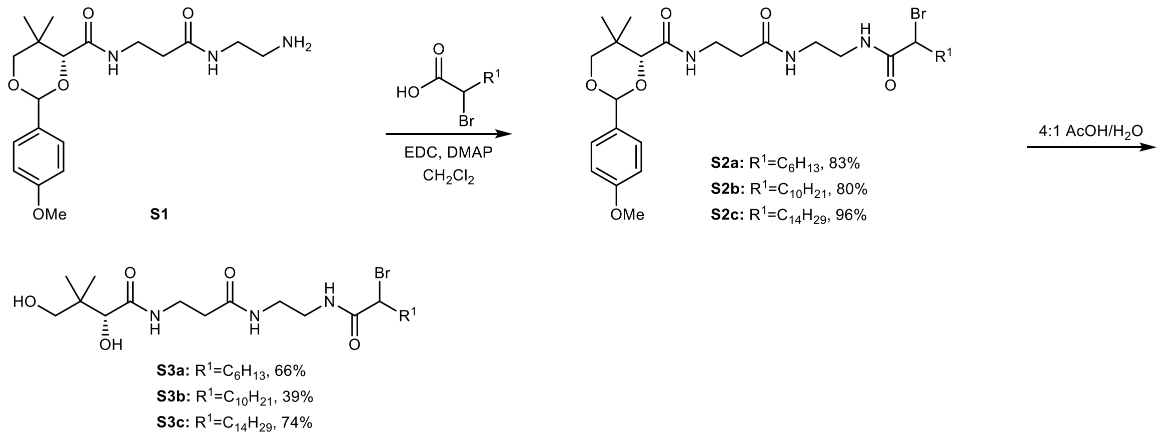

Synthesis of α-bromopantetheineamide crosslinkers. EDC=1-Ethyl-3-(3-dimethylaminopropyl)carbodiimide); DMAP=4-(dimethylamino)-pyridine; AcOH=acetic acid.

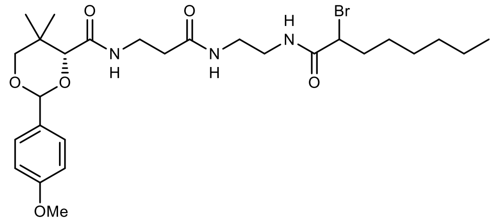

(4R)-N-(3-((2-(2-bromooctanamido)ethyl)amino)-3-oxopropyl)-2-(4-methoxy-phenyl)-5,5-dimethyl-1,3-dioxane-4-carboxamide (S2a). In a 10 mL pear-shaped flask, (4*R*)-*N*-(3-((2-aminoethyl)amino)-3-oxopropyl)-2-(4-methoxyphenyl)-5,5-dimethyl-1,3-dioxane-4-carboxamide **S1**[15] (100.8 mg, 0.2656 mmol, 1.0 equiv.), 2-bromooctanoic acid (69.6 μL, 0.3987 mmol, 1.5 equiv.), and 2.7 mL CH_2Cl_2 were added. To the solution was added EDC•HCl (85.2 mg, 0.4444 mmol, 1.7 equiv.) and DMAP (9.7 mg, 0.0794 mmol, 0.30 equiv.). After 18 h, the reaction was dissolved in 100 mL CH_2Cl_2 and washed with brine (10 mL). The organic phase was dried ($MgSO_4$), filtered, and concentrated by rotary evaporation. Purification by silica flash chromatography (39:1 → 19:1 CH_2Cl_2/MeOH) afforded **S2a** (128.7 mg, 83%) as a white solid.

TLC (CH_2Cl_2/MeOH, 19:1 v:v): R_f = 0.24; ^{1}H-NMR (400 MHz, $CDCl_3$): δ 7.50 (bs, 1H), 7.38 (d, *J* = 8.6 Hz, 2H), 7.30 (bs, 1H), 7.11 (t, *J* = 5.8 Hz, 1H), 6.90 – 6.82 (d, *J* = 8.3 Hz, 2H), 5.40 (s, 1H), 4.22–4.09 (m, 1H), 4.02 (s, 1H), 3.75 (s, 3H), 3.62 (q, *J* = 11.4 Hz, 2H), 3.51–3.40 (m, 2H), 3.36 (bs, 5H), 3.26 (bs, 3H), 3.03 (s, 3H), 2.35 (t, *J* = 6.3 Hz, 2H), 1.21 (bs, 6H), 1.04 (s, 3H), 1.01 (s, 3H), 0.81 (t, *J* = 6.5 Hz, 2H). ^{13}C-NMR (100 MHz, $CDCl_3$): δ 172.31, 170.31, 169.74, 160.15, 130.02, 127.49, 113.65, 101.26, 83.76, 78.32, 55.27, 50.25, 39.97, 38.91, 35.83, 35.79, 35.37, 35.20, 33.01, 31.48, 28.47, 27.23, 22.46, 21.76, 19.07, 13.99; (*m/z*): 606.31([M+Na]$^+$.

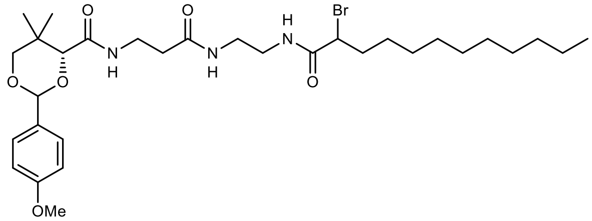

(4R)-N-(3-((2-(2-bromododecanamido)ethyl)amino)-3-oxopropyl)-2-(4-methoxyphenyl)-5,5-dimethyl-1,3-dioxane-4-carboxamide (S2b). Prepared as described for **S2a** from **S1** and 2-bromododecanoic acid to give **S2b** (146.6 mg, 80%) as a clear oil. TLC (CH_2Cl_2/MeOH, 19:1 v:v): R_f = 0.24; ^{1}H-NMR (400 MHz, $CDCl_3$): δ 7.43 (d, J = 8.1 Hz, 2H), 7.03 (s, 2H), 6.92 (d, J = 8.0 Hz, 2H), 6.61 (bs, 1H), 5.47 (s, 1H), 4.33–4.19 (m, 3H), 4.19–4.12 (m, 1H), 4.10 (s, 1H), 3.82 (s, 2H), 3.69 (q, J = 11.4 Hz, 2H), 3.59–3.51 (m, 2H), 3.37 (bs, 2H), 2.45 (t, J = 5.5 Hz, 2H), 1.99–1.88 (m, 2H),1.29–1.21 (m, 14H), 1.10 (d, J = 6.5 Hz, 6H), 0.92–0.82 (m, 5H). ^{13}C-NMR (100 MHz, $CDCl_3$): δ 172.48, 171.13, 170.61, 170.33, 160.26, 130.09, 127.59, 113.74, 101.39, 83.80, 78.42, 74.53, 69.15, 62.37, 62.02, 55.34, 50.68, 40.22, 39.20, 36.11, 35.56, 35.23, 34.97, 34.14, 33.13, 31.93, 31.77, 29.73, 29.60, 29.44, 29.36, 29.26, 29.11, 28.92, 27.38, 25.34, 24.88, 22.72, 21.87, 20.93, 20.74, 19.20, 14.17. ESI-MS (m/z): 663.39([M+Na]$^+$, 582.50 [M-Br+Na]$^+$.

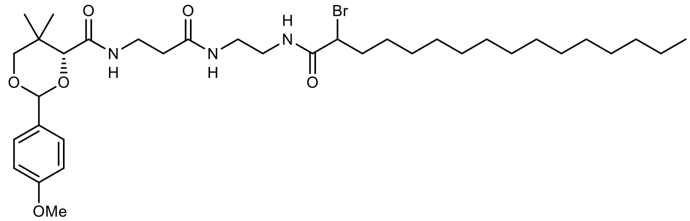

(4R)-N-(3-((2-(2-bromohexadecanamido)ethyl)amino)-3-oxopropyl)-2-(4-methoxyphenyl)-5,5-dimethyl-1,3-dioxane-4-carboxamide (S2c). Prepared as described for **S2a** from **S1** and 2-bromohexadecanoic acid to give **S2c** (178.4 mg, 96%) as a white solid. Characterization data were consistent with those previously reported.[16]

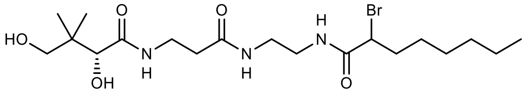

2-bromo-N-(2-(3-((R)-2,4-dihydroxy-3,3-dimethylbutanamido)propanamido)ethyl)-octanamide (S3a). In a 20 mL vial, **S2a** (128.7 mg, 0.2202 mmol, 1.0 equiv.) and 2.2 mL 4:1 AcOH/H_2O were added. After 5 h, the mixture was concentrated by rotary evaporation, and azeotroped from cyclohexane (3x10 mL) and benzene (3x10 mL). Purification by silica flash chromatography (19:1 → 4:1 CH_2Cl_2/MeOH) afforded **S3a** (65.8 mg, 66%) as a white solid.

TLC (CH_2Cl_2/MeOH, 9:1 v:v): R_f = 0.37; ^{1}H-NMR (400 MHz, $CDCl_3$): δ 7.35–7.28 (m, 1H), 7.11–7.01 (m, 1H), 6.71–6.64 (m, 1H), 6.61–6.55 (m, 1H), 4.37– 4.14 (m, 2H), 4.02 (d, J = 4.9 Hz, 1H), 3.78–3.58 (m, 1H), 3.57–3.52 (m, 1H), 3.48–3.23 (m, 4H), 2.45 (q, J = 6.9, 6.4 Hz, 2H), 2.14–1.90 (m, 2H), 1.51–1.37 (m, 2H), 1.36–1.23 (m, 6H), 1.03 (s, 3H), 0.94

(s, 3H), 0.88 (t, *J* = 6.6 Hz, 3H). ^{13}C-NMR (100 MHz, $CDCl_3$): δ 174.47, 172.65, 170.78, 70.62, 50.38, 39.95, 39.35, 36.07, 35.50, 31.65, 28.66, 27.43, 22.63, 21.28, 20.85, 14.17.ESI-MS (*m/z*): 488.28 ($[M+Na]^+$.

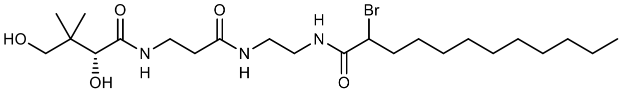

2-bromo-N-(2-(3-((R)-2,4-dihydroxy-3,3-dimethylbutanamido)propanamido)ethyl)-dodecanamide (S3b). Prepared as described for **S3a** from **S2b**, to give **S3b** (47.1 mg, 39%) as a white solid.

TLC (CH_2Cl_2/MeOH, 9:1 v:v): R_f = 0.46; ^{1}H-NMR (400 MHz, $CDCl_3$): δ 7.63–7.48 (m, 2H), 7.32 (s, 1H), 4.25 (bs, 1H), 3.99 (s, 1H), 3.65–3.53 (m, 1H), 3.52–3.38 (m, 5H), 3.37–3.25 (m, 2H), 2.44 (s, 2H), 1.98 (d, *J* = 31.1 Hz, 2H), 1.42 (s, 1H), 1.23 (bs, 16H), 0.96 (s, 3H), 0.91 (s, 3H), 0.88–0.82 (m, 3H); ^{13}C-NMR (100 MHz, $CDCl_3$): δ 174.41, 172.66, 170.74, 70.76, 50.56, 40.07, 39.36, 36.10, 35.54, 32.00, 29.68, 29.53, 29.43, 29.03, 27.51, 22.78, 21.38, 20.79, 14.24. ESI-MS (*m/z*): 544.31$[M+Na]^+$, 464.40 $[M-Br+Na]^+$, 520.17 $[M-H]^-$.

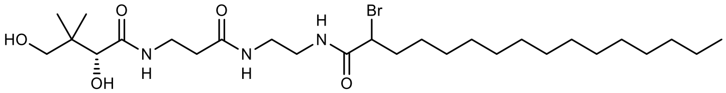

2-bromo-N-(2-(3-((R)-2,4-dihydroxy-3,3-dimethylbutanamido)propanamido)ethyl)-hexadecanamide (S3c). Prepared as described for **S3a** from **S2c**, to give **S3c** (110.4 mg, 74%) as a white solid. Characterization data were consistent with those previously reported.[3]

Supplementary Figure 40. NMR characterization of S2a

1H NMR (400 MHz) Spectrum of S2a

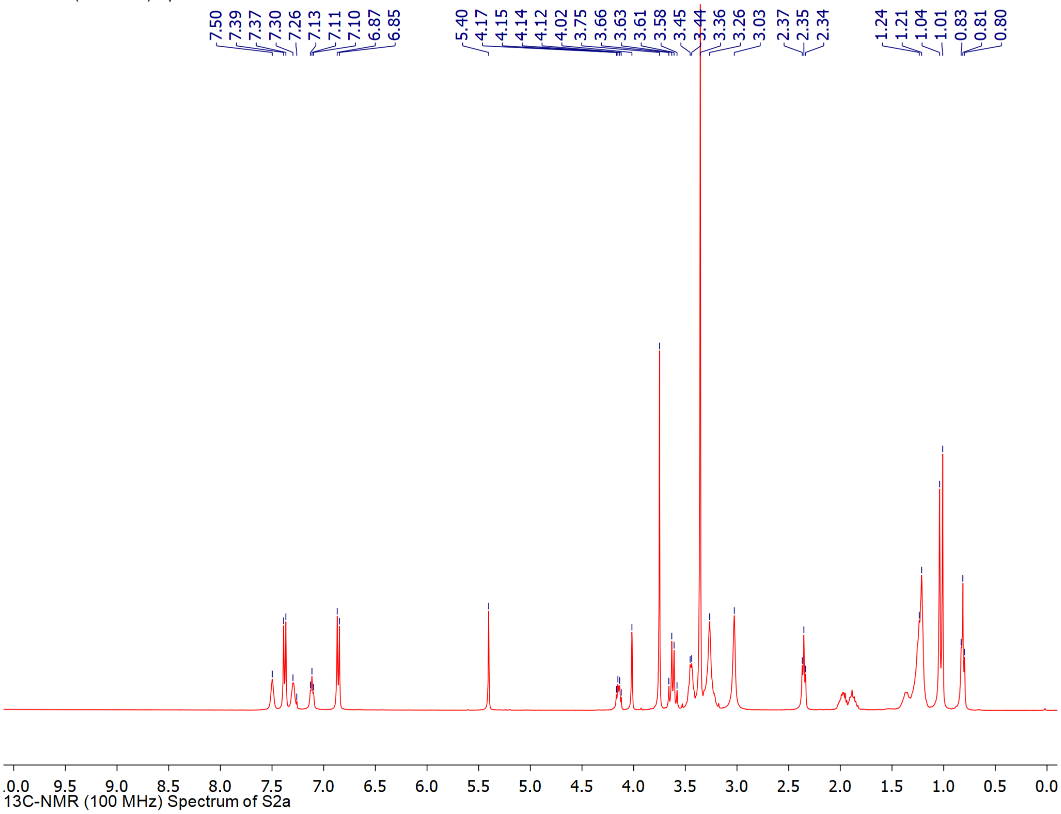

13C-NMR (100 MHz) Spectrum of S2a

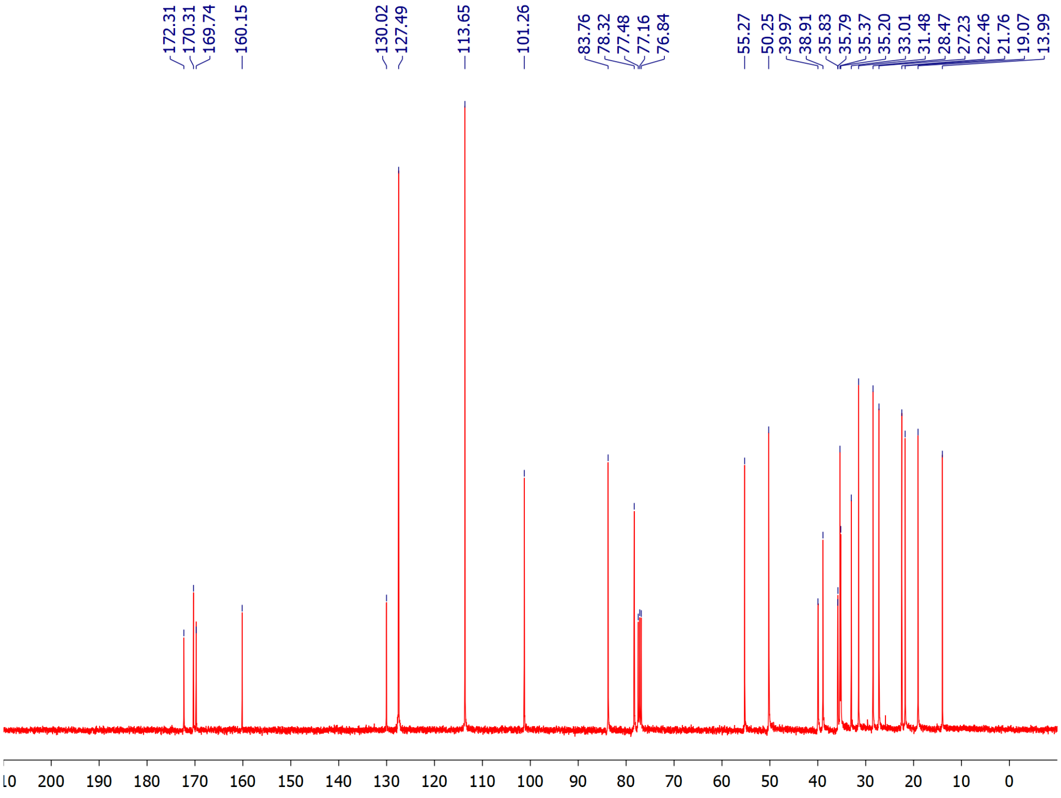

Supplementary Figure 41. NMR characterization of S2b

1H-NMR (400 MHz) Spectrum of S2b

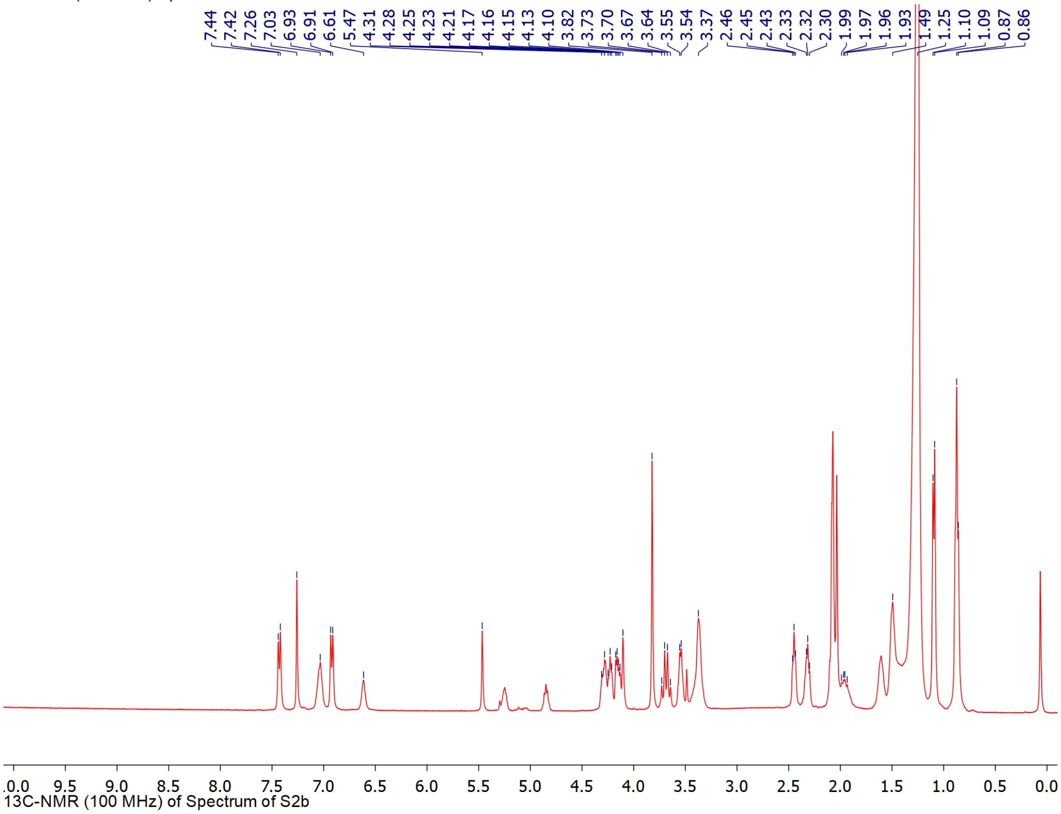

13C-NMR (100 MHz) of Spectrum of S2b

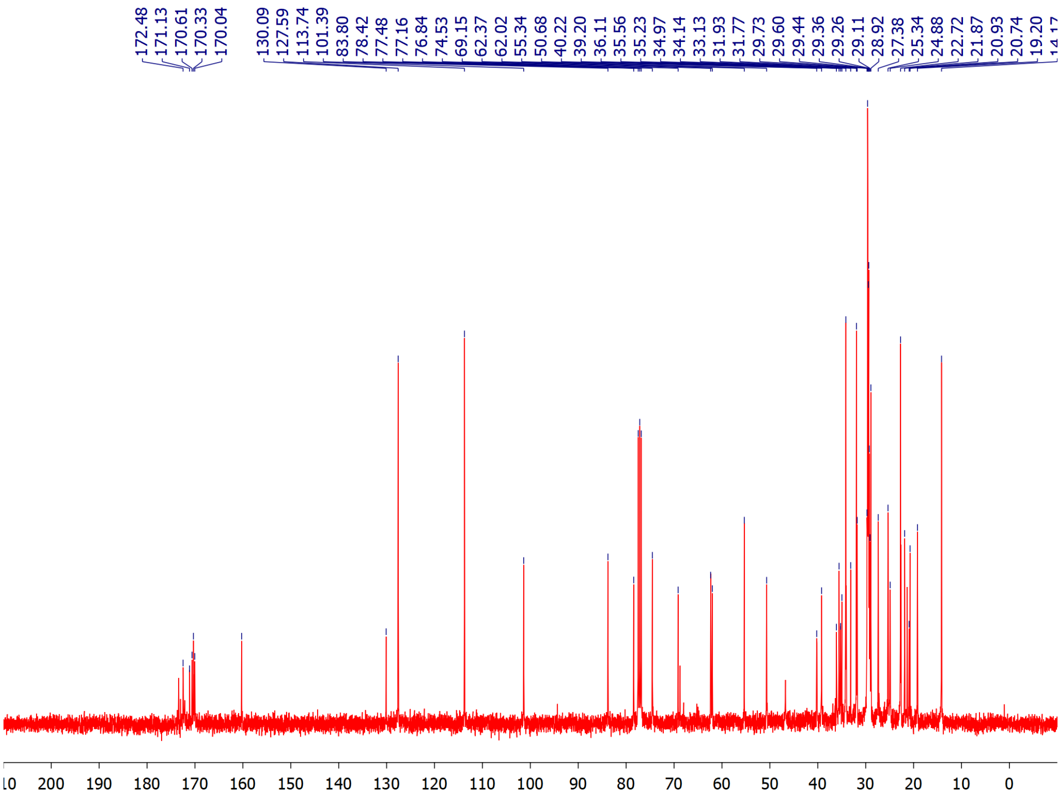

Supplementary Figure 42. NMR characterization of S3a

1H-NMR (400 MHz) Spectrum of S3a

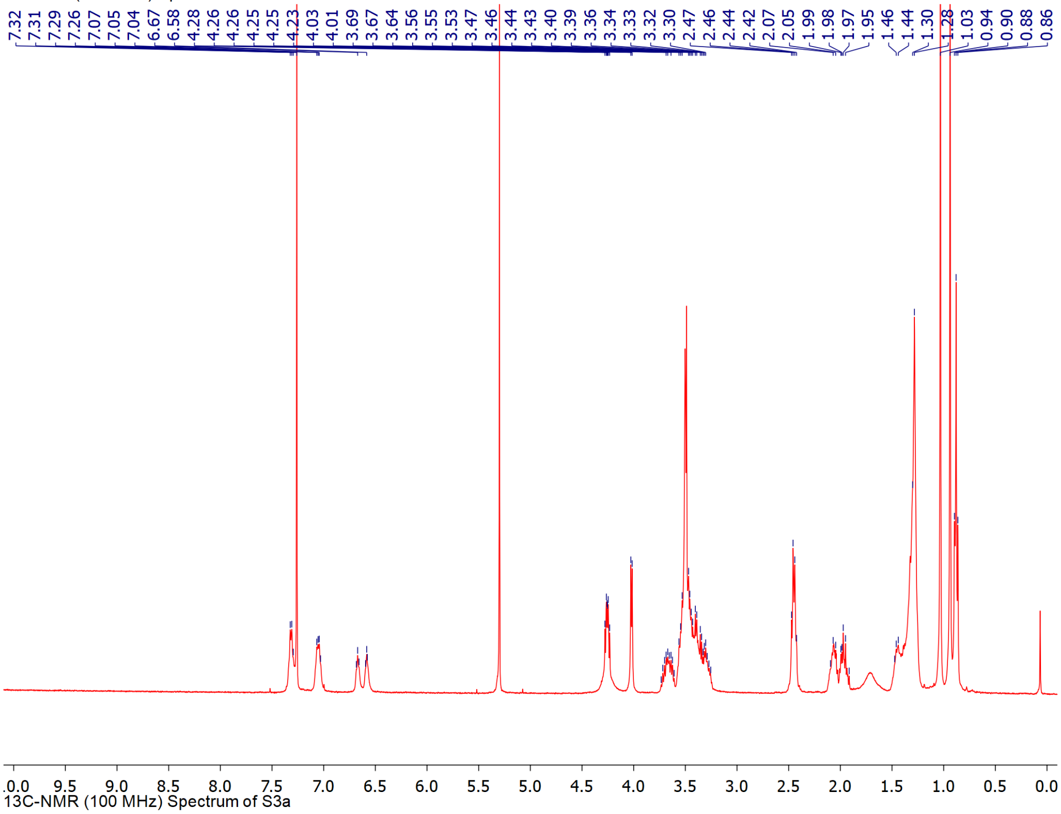

13C-NMR (100 MHz) Spectrum of S3a

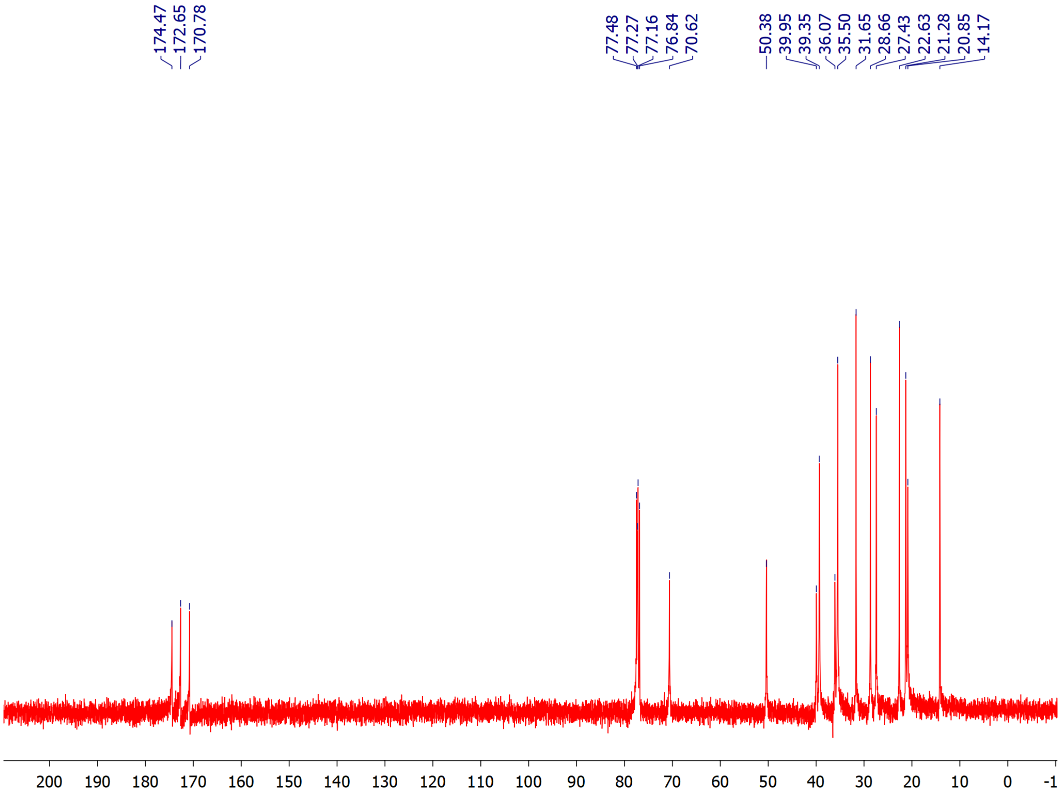

Supplementary Figure 43. NMR characterization of S3b

1H-NMR (400 MHz) Spectrum of S3b

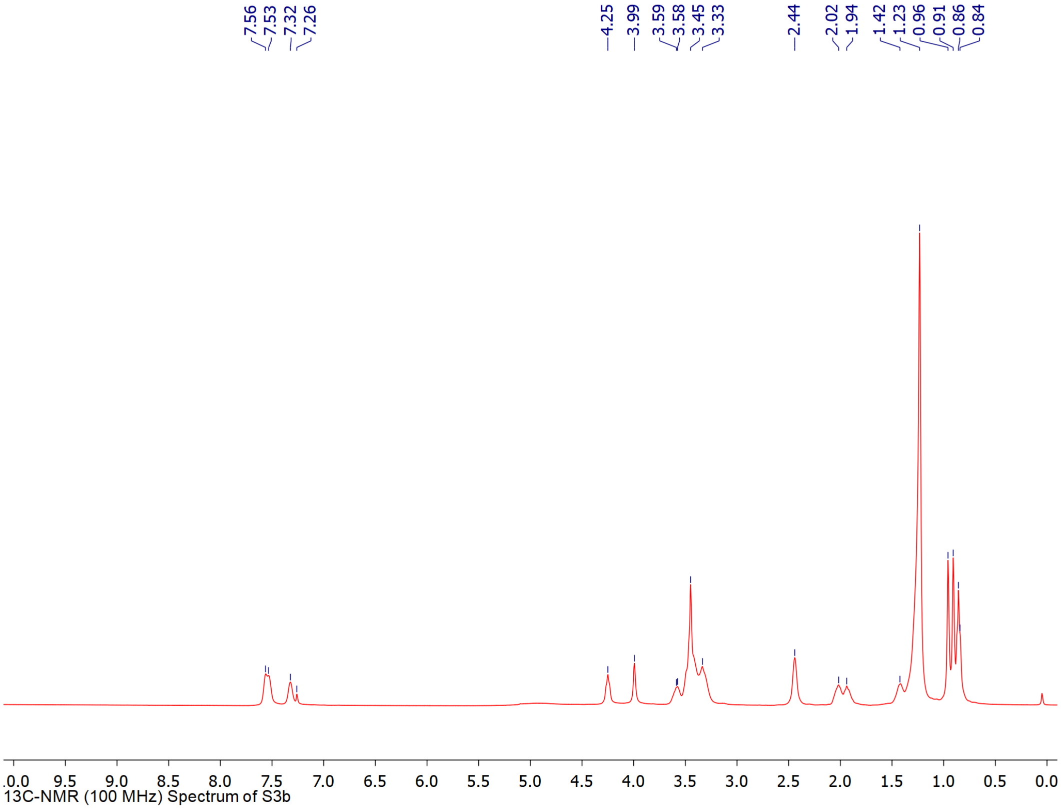

13C-NMR (100 MHz) Spectrum of S3b

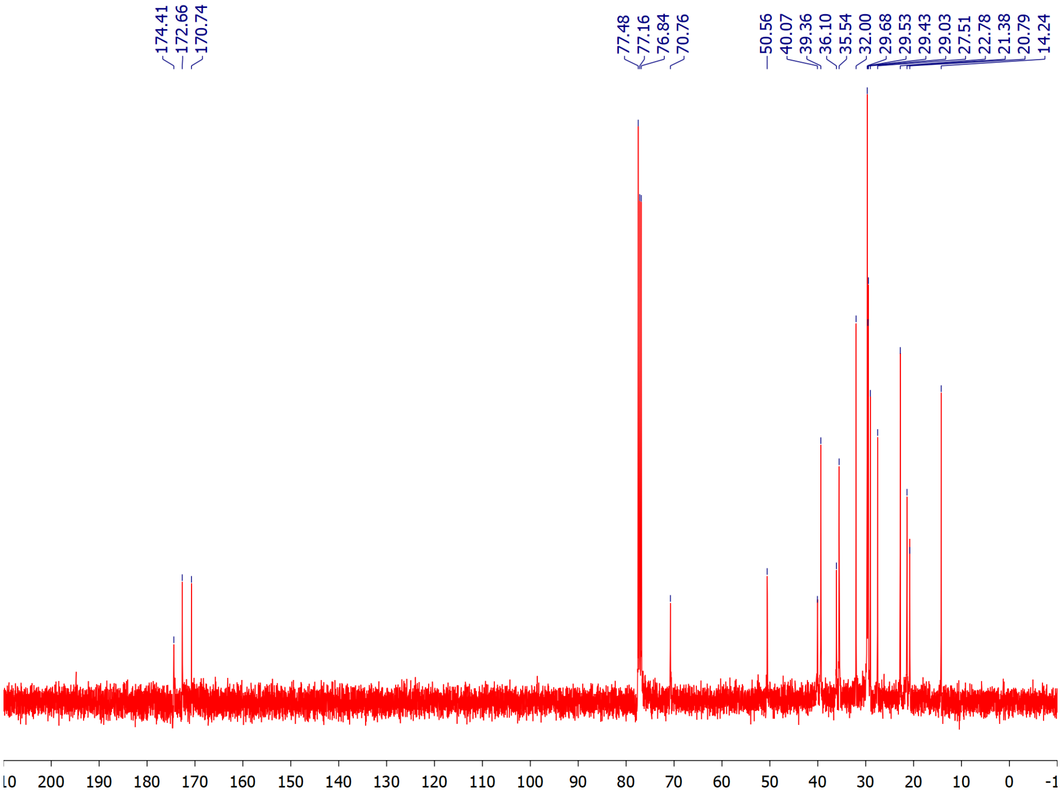

Supplementary References

1. Olsen, J. G., Kadziola, A., von Wettstein-Knowles, P., Siggaard-Andersen, M. & Larsen, S. Structures of β-Ketoacyl-Acyl Carrier Protein Synthase I Complexed with Fatty Acids Elucidate its Catalytic Machinery. *Structure* **9,** 233–243 (2001).
2. D'Agnolo, G., Rosenfeld, I. S. & Vagelos, P. R. Multiple forms of beta-ketoacyl-acyl carrier protein synthetase in Escherichia coli. *J. Biol. Chem.* **250,** 5289–5294 (1975).
3. Garwin, J., Klages, A. & Cronan, J. Structural, enzymatic, and genetic studies of beta-ketoacyl-acyl carrier protein synthases I and II of Escherichia coli. *J. Biol. Chem.* **255,** 11949–11956 (1980).
4. de Mendoza, D. & Cronan, J. E. Thermal regulation of membrane lipid fluidity in bacteria. *Trends Biochem. Sci.* **8,** 49–52 (1983).
5. Edwards, P., Sabo Nelsen, J., Metz, J. G. & Dehesh, K. Cloning of the fabF gene in an expression vector and in vitro characterization of recombinant fabF and fabB encoded enzymes from Escherichia coli. *FEBS Lett.* **402,** 62–66 (1997).
6. Beld, J., Lee, D. J. & Burkart, M. D. Fatty acid biosynthesis revisited: structure elucidation and metabolic engineering. *Mol. Biosyst.* **11,** 38–59 (2015).
7. Heath, R. J. & Rock, C. O. Roles of the FabA and FabZ beta-hydroxyacyl-acyl carrier protein dehydratases in Escherichia coli fatty acid biosynthesis. *J Biol Chem* **271,** (1996).
8. Feng, Y. & Cronan, J. E. Escherichia coli unsaturated fatty acid synthesis: Complex transcription of the FabA gene and in vivo identification of the essential reaction catalyzed by FabB. *J. Biol. Chem.* **284,** 29526–29535 (2009).
9. Magnuson, K., Jackowski, S., Rock, C. O. & Cronan, J. E. Regulation of fatty acid biosynthesis in Escherichia coli. *Microbiol. Rev.* **57,** 522 LP-542 (1993).
10. Bloch, K. in (ed. Boyer, P. D. B. T.) **5,** 441–464 (Academic Press, 1971).

11. Garwin, J. L. & Cronan, J. E. Thermal modulation of fatty acid synthesis in Escherichia coli does not involve de novo enzyme synthesis. *J. Bacteriol.* **141,** 1457–1459 (1980).

12. Gora, A., Brezovsky, J. & Damborsky, J. Gates of enzymes. *Chem. Rev.* **113,** 5871–5923 (2013).

13. Wang, J. *et al.* Platensimycin is a selective FabF inhibitor with potent antibiotic properties. *Nature* **441,** 358–361 (2006).

14. Gottlieb, H. E., Kotlyar, V. & Nudelman, A. NMR Chemical Shifts of Common Laboratory Solvents as Trace Impurities. *J. Org. Chem.* **62,** 7512–7515 (1997).

15. Meier, J. L. & Burkart, M. D. Chapter 9. Synthetic probes for polyketide and nonribosomal peptide biosynthetic enzymes. *Methods Enzymol.* **458,** 219–254 (2009).

16. Blatti, J. L. *et al.* Manipulating fatty acid biosynthesis in microalgae for biofuel through protein-protein interactions. *PLoS One* **7,** 1–12 (2012).
